# Inhibition of key DNA double strand break repair protein kinases enhances radiosensitivity of head and neck cancer cells to X-ray and proton irradiation

**DOI:** 10.1038/s41420-024-02059-3

**Published:** 2024-06-12

**Authors:** Maria Rita Fabbrizi, Thomas J. Doggett, Jonathan R. Hughes, Emma Melia, Elizabeth R. Dufficy, Rhianna M. Hill, Amalia Goula, Ben Phoenix, Jason L. Parsons

**Affiliations:** 1 Institute of Cancer and Genomic Sciences, University of Birmingham, Edgbaston, UK; 2 Department of Molecular and Clinical Cancer Medicine, University of Liverpool, Liverpool, UK; 3 School of Physics and Astronomy, University of Birmingham, Edgbaston, UK

**Keywords:** Head and neck cancer, Cell death

## Abstract

Ionising radiation (IR) is widely used in cancer treatment, including for head and neck squamous cell carcinoma (HNSCC), where it induces significant DNA damage leading ultimately to tumour cell death. Among these lesions, DNA double strand breaks (DSBs) are the most threatening lesion to cell survival. The two main repair mechanisms that detect and repair DSBs are non-homologous end joining (NHEJ) and homologous recombination (HR). Among these pathways, the protein kinases ataxia telangiectasia mutated (ATM), ataxia telangiectasia and Rad3-related (ATR) and the DNA dependent protein kinase catalytic subunit (DNA-Pkcs) play key roles in the sensing of the DSB and subsequent coordination of the downstream repair events. Consequently, targeting these kinases with potent and specific inhibitors is considered an approach to enhance the radiosensitivity of tumour cells. Here, we have investigated the impact of inhibition of ATM, ATR and DNA-Pkcs on the survival and growth of six radioresistant HPV-negative HNSCC cell lines in combination with either X-ray irradiation or proton beam therapy, and confirmed the mechanistic pathway leading to cell radiosensitisation. Using inhibitors targeting ATM (AZD1390), ATR (AZD6738) and DNA-Pkcs (AZD7648), we observed that this led to significantly decreased clonogenic survival of HNSCC cell lines following both X-ray and proton irradiation. Radiosensitisation of HNSCC cells grown as 3D spheroids was also observed, particularly following ATM and DNA-Pkcs inhibition. We confirmed that the inhibitors in combination with X-rays and protons led to DSB persistence, and increased micronuclei formation. Cumulatively, our data suggest that targeting DSB repair, particularly via ATM and DNA-Pkcs inhibition, can exacerbate the impact of ionising radiation in sensitising HNSCC cell models.

## Introduction

Head and neck cancers (often referred to as head and neck squamous cell carcinoma (HNSCC)) usually develop in or around the throat, larynx, nose, sinuses, and mouth. HNSCC is the eighth most common cancer type in the UK with ~12,000 incidences per year [1] and the seventh most common cancer globally with ~890,000 new cases per year [2]. Tobacco and alcohol use together with high-risk (type-16/18) human papillomavirus (HPV) infection are the common risk factors. Currently, surgery, radiotherapy and chemotherapy are the main treatments for HNSCC. The major goal and outcome of radiotherapy is to specifically target and damage the DNA within the cancer cells, up to a point where the cells are unable to recover and therefore undergo cell death. In this regard, proton beam therapy (PBT) is increasingly being utilised for cancer treatment where it displays significant advantages over conventional X-ray radiotherapy, particularly the precise delivery of the radiation dose to the tumour and thus sparing of the normal tissues and organs at risk [3]. This is particularly important when treating tumours that are close to a critical part of the body, such as the spinal cord [4]. Highly complex brain, HNSCC and sarcomas are currently being treated with PBT due to their poor outcome when treated with X-rays. Regardless of whether X-rays or PBT are utilised, the introduction of DNA double strand breaks (DSBs) and complex DNA damage containing multiple lesions in close proximity are considered the most lethal lesions contributing to ionising radiation-induced cell death. Nevertheless, these DNA lesions activate the cellular DNA damage response (DDR), which is a coordinated network of proteins that act through protein post-translational modifications to sense and repair the DNA damage to restore DNA integrity [5, 6], and can consequently drive tumour radioresistance.

Ataxia telangiectasia mutated (ATM), ataxia telangiectasia and Rad-3 related (ATR) and DNA-dependent protein kinase catalytic subunit (DNA-Pkcs) are protein kinases that are fundamental in the signalling and repair of DNA DSBs. The first step is achieved through recognition of the damage and stimulation of the phosphorylation of the histone H2AX variant (forming γH2AX). The binding of the Ku70/80 heterodimer to the DSB ends along with DNA-Pkcs stimulates NHEJ, whereas DNA end resection through the action of the MRN complex enables HR which is restricted to specific stages of the cell cycle [7, 8]. Interestingly, overexpression of ATR has been observed in a subset of oral squamous cell carcinoma (OSCC) cell lines [9], whereas upregulation of DNA-Pkcs has been associated with radioresistance in cancers of the nasopharynx [10] and oral cavity [11]. Conversely, reduced expression of ATM has been associated with the poor outcome of laryngeal and pharyngeal cancer patients, and loss of one ATM allele or low ATM expression is suggested to confer a reduced cellular DDR and radioresistance of HNSCC cells [12, 13].

Due to their paramount importance in resolving DNA DSB damage, ATM, ATR and DNA-Pkcs have been increasingly investigated to confirm whether their inhibition could improve the impact of X-ray irradiation in decreasing HNSCC cell survival [14–16]. Using HNSCC cell lines (HN4 and HN5), treatment with the DNA-Pkcs inhibitor KU-0060648 increased cellular radiosensitivity associated with increased radiation-induced RAD51 and γH2AX foci, along with G2/M cell cycle arrest [17]. These results were confirmed by another study using two different DNA-Pkcs inhibitors (KU-57788 and IC87361) that observed increased radiosensitivity of four HNSCC cell lines (UT-SCC1A, UT-SCC42B, UTT-SCC-4C and UT-SCC110B) [18]. An inhibitor of ATM, GSK635416A, was observed to have a radiosensitising effect on HNSCC cells (UT-SCC24A, UT-SCC36 and UTT-SCC4) that was apparently greater than that observed with PARP (olaparib) or EGFR (cetuximab) inhibition [19]. There is also evidence that ATR inhibition through AZD6738 and VE-821 is able to radiosensitise various HNSCC cell lines [17, 20, 21]. A previous study conducted in our lab also demonstrated that the clonogenic survival and growth of 3D spheroids were significantly reduced in HNSCC cell lines treated with ATM (KU-55933), ATR (VE-821) and DNA-Pkcs inhibitors (KU-57788) in the presence of X-ray radiation, but also following PBT [22]. Here we observed that inhibition of ATM and DNA-Pkcs appeared to produce the most profound radiosensitisation of HNSCC following both radiation types. Interestingly, studies suggest that the biological impact of PBT versus X-rays may be different due to the different particle type but also differences in ionisation density (linear energy transfer (LET)), indicating more of a preference on either NHEJ or HR for DNA DSB repair in response to PBT [3]. Our previous study would contradict this observation; however, there are currently no other studies that have systematically compared the effect of ATM, ATR and DNA-Pkcs inhibitors in HNSCC cell lines following PBT and X-ray irradiation. This evidence is of paramount important in order to devise the optimal strategies using these different types of radiotherapy to promote enhanced tumour cell radiosensitivity.

Herein, we have characterised the comparative impact of potent and specific inhibitors of ATM, ATR and DNA-Pkcs on the response of several HPV-negative HNSCC cells to both X-rays and PBT. Our results demonstrate that inhibiting these protein kinases significantly reduces the growth of HNSCC models in both 2D and 3D, causing persistent DNA DSB formation and chromosomal instability. Collectively, our findings demonstrate that these inhibitors should be exploited further in more advanced HNSCC models for the effective treatment of patients following either X-ray radiotherapy or PBT.

## Results

### Targeting ATM, ATR and DNA-Pkcs enhances sensitivity of HNSCC cells to X-rays

**Fig. 1:**
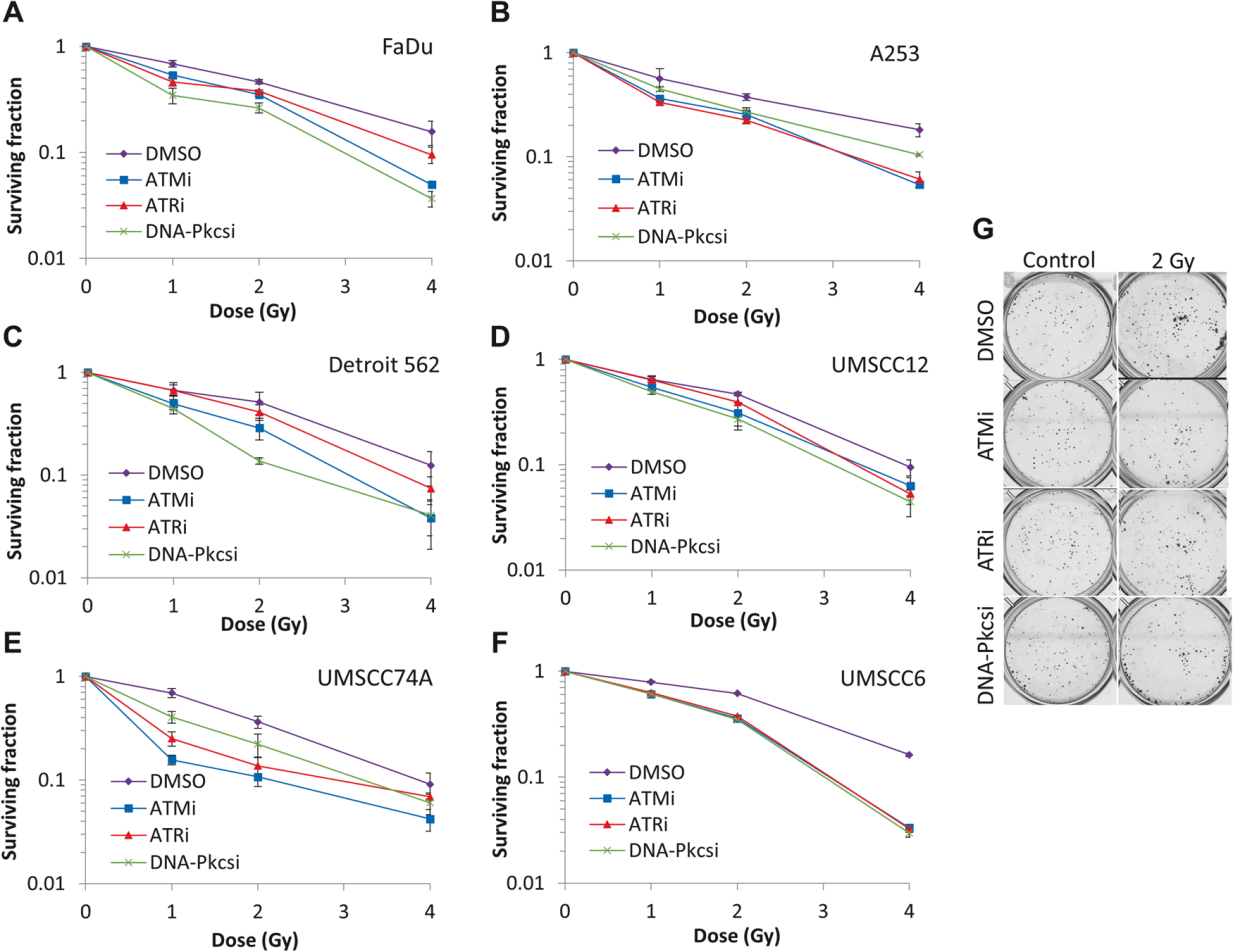
Inhibition of ATM, ATR and DNA-Pkcs leads to increased radiosensitivity of HNSCC cells in response to X-ray radiation. **A** FaDu, **B** A253, **C** Detroit 562, **D** UMSCC12, **E** UMSCC74A and **F** UMSCC6 cells were pretreated with inhibitors targeting ATM (10 nM), ATR (1 µM) and DNA-Pkcs (1 µM) or DMSO as a vehicle only control, for 1 h prior to irradiation. Cells were then irradiated with increasing doses of X-rays, and clonogenic survival of cells was analysed from three biologically independent experiments. Shown is the mean surviving fraction±S.E. **G** Representative images of FaDu colonies formed from unirradiated (Control) cells, and those following a 2 Gy dose of X-rays (double the numbers of cells seeded).

**Fig. 2:**
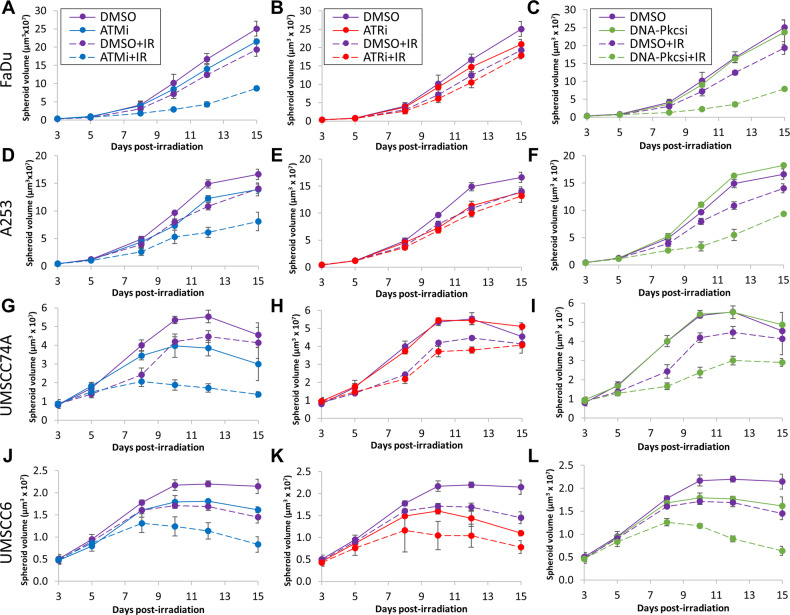
Inhibition of ATM, ATR and DNA-Pkcs leads to reduced growth of HNSCC 3D spheroids following X-ray radiation. Spheroids were allowed to develop for 48 h in ultra-low attachment plates, and then treated with inhibitors targeting ATM, ATR, or DNA-Pkcs (all 0.2 µM) or DMSO as a vehicle only control for 1 h. Spheroids were then unirradiated, or irradiated with 1 Gy X-rays and growth of **A**–**C** FaDu, **D**–**F** A253, **G**–**I** UMSCC74A and **J**–**L** UMSCC6 spheroids measured by microscopy up to 15 days post-seeding. Shown is the mean spheroid volume ± S.E., analysed from three biologically independent experiments.

We first analysed the impact of targeting the major protein kinases involved in DNA DSB repair using potent and characterised inhibitors (ATMi, AZD1390; ATRi, AZD6738; DNA-Pkcsi, AZD7648) on the survival of HNSCC cell lines post X-ray irradiation. The HNSCC cell lines used in this study demonstrate differential expression levels of ATM, ATR and DNA-Pkcs as shown by immunoblotting (Supplementary Figs. 1 and 2). The specific dose used for each inhibitor was determined through the suppression of kinase activity on UMSCC74A cells via immunoblotting (10 nM for ATMi, 1 µM for DNA-Pkcsi; Supplementary Figs. 3A–C and 4A–C). Due to the relatively high toxicity of the ATR inhibitor, AZD6738, a lower concentration (1 µM) had to be used for clonogenic survival assays which only partially suppressed the activity of the protein kinase. In combination with X-ray irradiation, we demonstrate that there was a significant reduction in clonogenic survival of HNSCC cells in the presence of either ATMi, ATRi or DNA-Pkcsi versus the DMSO control (Fig. 1A–G; see also Supplementary Fig. 5 and Supplementary Table 1). Dose enhancement ratios calculated at 50% survival (DER_50_) ranged from 1.28-2.07 (ATMi), 1.15–2.49 (ATRi) and 1.41–2.86 (DNA-Pkcsi) with each cell line responding differently. Consistently, the majority of the cell lines were more susceptible to DNA-Pkcs and ATM inhibition, whereas some cells (particularly Detroit 562 and UMSCC12) were less radiosensitised in the presence of ATRi. We subsequently analysed the impact of the DNA DSB repair inhibitors on the radiosensitivity of HNSCC cells grown as 3D spheroids, which more accurately reflect the structure and environment of the original tumour. Spheroids grew differently in volume in the absence of any treatments as we previously observed [22], with FaDu and A253 growing ~16–25-fold after 15 days post-seeding (Fig. 2A, D and Supplementary Fig. 6A, B), whereas UMSCC74A and UMSCC6 only grew ~2.2–5.5-fold in volume (Fig. 2G, J and Supplementary Fig. 6C, D). Nevertheless, we demonstrated that the combination of ATMi (0.2 µM) plus X-ray radiation was significantly effective in suppressing spheroid growth compared to radiation alone in all the cell lines analysed (Fig. 2A, D, G, J; see also Supplementary Fig. 6A–D and Supplementary Tables 2 and 3; DER = 1.84–4.33). In contrast, ATRi with X-ray radiation showed little enhancement in cellular radiosensitivity, apart from in UMSCC6 cells (Fig. 2B, E, H, J; see also Supplementary Fig. 6A–D and Supplementary Tables 2 and 3; DER = 1.10–1.98). Similar to ATMi, DNA-Pkcsi was able to strongly and significantly sensitise all the spheroid models to X-ray irradiation (Fig. 2C, F, I, L; see also Supplementary Fig. 6A–D and Supplementary Tables 2 and 3; DER = 1.69–3.70). Using a higher concentration of the inhibitors (1 µM), this led to increased toxicity of the drugs alone in the spheroid model particularly with ATRi, although the radiosensitising effects of DNA-Pkcsi and ATMi was still evident (Supplementary Fig. 7A–L). Therefore, these results suggest that inhibition of particularly ATM and DNA-Pkcs are effective in reducing the survival of both 2D and 3D cell models of HNSCC following X-ray radiation.

### ATM, ATR and DNA-Pkcs inhibition causes increased DSB persistence and chromosomal aberrations

**Fig. 3:**
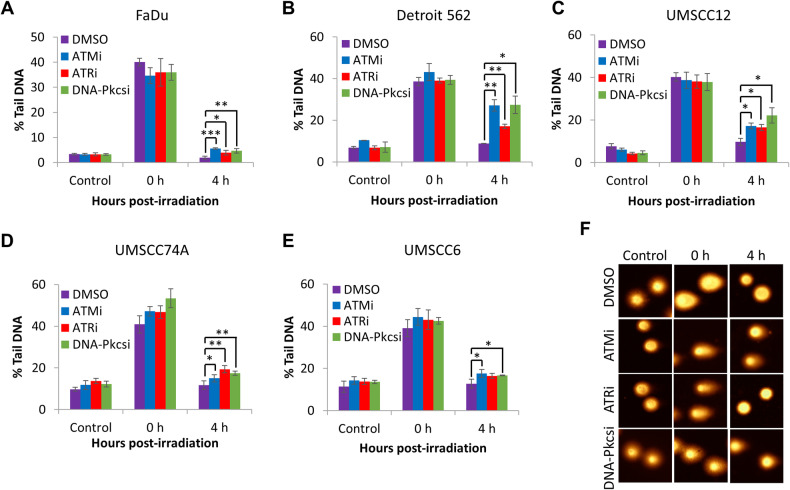
ATM, ATR and DNA-Pkcs are required for efficient repair of DSBs induced by X-ray radiation. **A** FaDu, **B** Detroit 562, **C** UMSCC12, **D** UMSCC74A and **E** UMSCC6 cells were treated with inhibitors targeting ATM (10 nM), ATR (1 µM) and DNA-Pkcs (1 µM) or DMSO as a vehicle only control for 1 h prior to irradiation. Cells were treated with 4 Gy X-rays and DNA DSB damage measured at various time points post-IR by the neutral comet assay. Shown is the mean % tail DNA ± S.D. *p < 0.05, **p < 0.02, ***p < 0.001 as analysed by a one-sample *t* test.

**Fig. 4:**
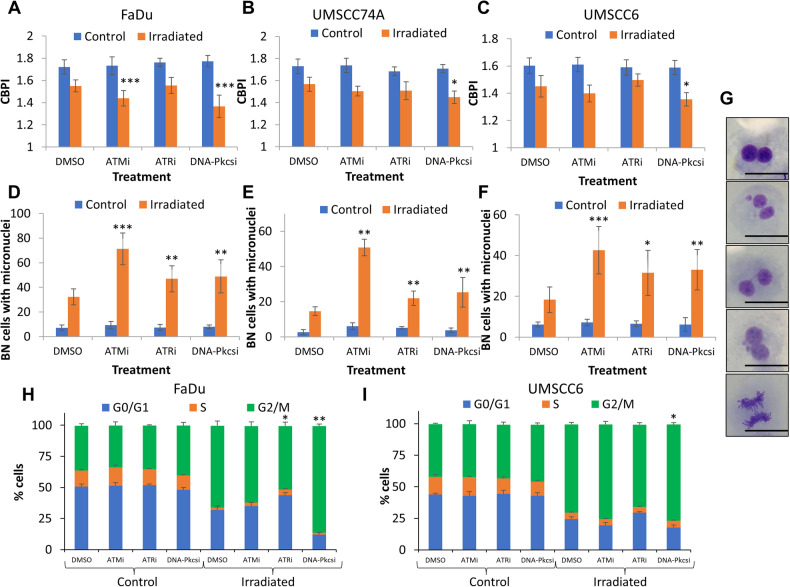
ATM, ATR and DNA-Pkcs inhibition induces chromosomal aberrations in HNSCC cells after X-ray irradiation. **A** FaDu, **B** UMSCC74A, and **C** UMSCC6 cells were treated with inhibitors targeting ATM (10 nM), ATR (1 µM) and DNA-Pkcs (1 µM) or DMSO as a vehicle only control for 1 h and then treated with X-rays (2 Gy). Proliferation index and micronuclei frequency was then scored by the cytokinesis-block micronuclei assay. Shown is the **A**–**C** mean CBPI ± S.E. and the **D**–**F** micronuclei frequency calculated as the mean binucleated with micronuclei scored in 1000 binucleated cells ± S.E. *p < 0.05, **p < 0.02, ***p < 0.01 as analysed by a one-sample *t* test comparing the drug plus irradiation sample versus the DMSO irradiated control. **G** Representative images of (from top to bottom) a binucleated cell, binucleated with micronuclei cell, nuclear bridge, nuclear buds and mitotic cells from FaDu cells. The scale bar indicated is 20 µm. Alternatively following inhibitor treatment, **H** FaDu and **I** UMSCC6 cells were subject to 10 Gy X-rays and cell cycle analysis performed 24 h post-irradiation using flow cytometry. Shown is the mean number of cells in each cell cycle phase ± S.E. *p < 0.05, **p < 0.01 as analysed by a one-sample *t* test comparing the G2/M population in the drug plus irradiation sample versus the DMSO irradiated control.

Using the neutral comet assay to directly detect DNA DSBs, we observed that treatment of HNSCC cells with the inhibitors alone had no significant impact on endogenous DSB levels (Fig. 3A–F; see Control). In combination with X-ray irradiation, inhibition of ATM, ATR and DNA-Pkcs was found not to enhance the levels of DSBs immediately post-irradiation (time 0 h); however, this significantly reduced the efficiency of DSB repair in comparison to DMSO control cells 4 h post-irradiation (Fig. 3A–F). DNA-Pkcsi and ATMi appeared to be the most effective in delaying DSB resolution, particularly in FaDu, Detroit 562 and UMSCC12 cells, whereas ATRi appeared to be comparatively less effective. We then analysed the effect of kinase inhibition on three HNSCC cell lines in terms of anti-proliferative ability and frequency of chromosomal abnormalities. Proliferation index, measured as CBPI, was expectedly reduced in all three HNSCC cell lines after treatment with X-rays (2 Gy). We furthermore observed in all the HNSCC cell lines that DNA-Pkcs inhibition in combination with X-rays caused a significant reduction in proliferation index when compared to DMSO with radiation (Fig. 4A–C). However, ATRi with X-ray radiation did not block proliferation in a statistically significant manner and ATMi only significantly reduced proliferation in FaDu cells (Fig. 4A–C). Interestingly, micronuclei frequency was found to be significantly elevated with all inhibitor-radiation combinations in all HNSCC cell lines, compared to the DMSO plus X-ray treated cells (Fig. 4D–G). Results on chromosome rearrangements, detected as nucleoplasmic bridges, and frequencies of nuclear buds as a biomarker of amplified DNA elimination, are summarised (Supplementary Table 4), while apoptotic and necrotic cells were also scored (Supplementary Table 5). In brief, there was only a significant increase in nucleoplasmic bridges and nuclear buds in UMSCC74A cells treated with ATMi in combination with X-rays. Also, only a significant increase in necrotic cells was observed in UMSCC74A cells treated with the combination of radiation with either ATMi or DNA-Pkcsi. We also investigated whether the inhibitors caused chromosome segregation anomalies rather than clastogenic effects. Here we observed a statistically significant increase in aneuploidogenic damage in UMSCC74A and UMSCC6 cells after treatment with either ATMi or DNA-Pkcsi in combination with X-ray radiation, compared to DMSO control irradiated cells (Supplementary Fig. 8A–C). Taken together, our results suggest that inhibition of ATM, ATR or DNA-Pkcs causes a significant increase in chromosomal aberrations post-irradiation in HNSCC cells. Finally, we also analysed the progression of FaDu and UMSCC6 cells through the cell cycle post-irradiation. As expected, X-ray irradiation (at a high 10 Gy dose in order to induce significant cell cycle checkpoint activation) caused an accumulation of cells in the G2/M phase compared to the unirradiated cells (Fig. 4H, I). No effects on cell cycle progression were observed following treatment with the kinase inhibitors alone. Treatment with ATMi in combination with X-ray radiation displayed no significant difference in G2/M accumulation of HNSCC cells compared to the radiation alone. In contrast, the combination of ATRi with X-rays caused a reduction of cells in G2/M phase (statistically significant in FaDu cells), while DNA-Pkcs inhibition led to a statistically significant accumulation of both FaDu and UMSCC6 cells in the G2/M phase (Fig. 4H, I).

### Growth and survival of HNSCC cells is reduced in the presence of inhibitors of ATM, ATR and DNA-Pkcs following PBT

**Fig. 5:**
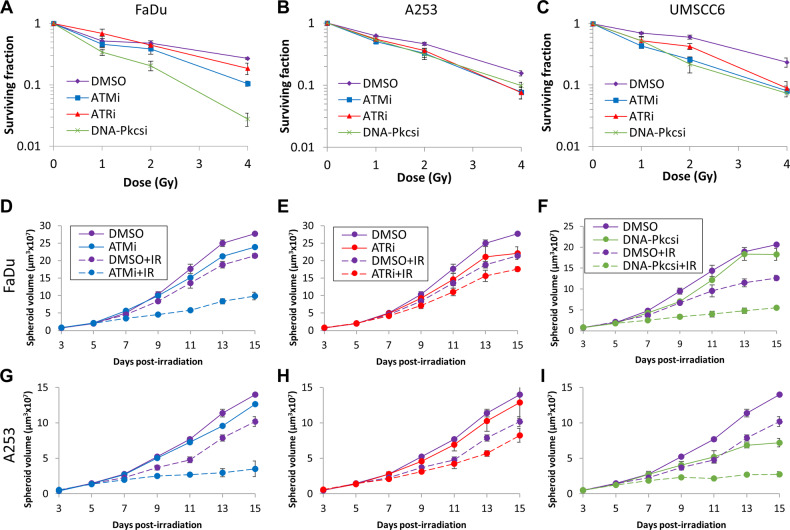
Inhibition of ATM, ATR and DNA-Pkcs leads to increased radiosensitivity of HNSCC cells in response to PBT. **A** FaDu, **B** A253 and **C** UMSCC6 cells were pretreated with inhibitors targeting ATM (10 nM), ATR (1 µM) and DNA-Pkcs (1 µM) or DMSO as a vehicle only control, for 1 h prior to irradiation. Cells were then irradiated with increasing doses of PBT, and clonogenic survival of cells was analysed from three biologically independent experiments. Shown is the mean surviving fraction±S.E. Alternatively, **D**–**F** FaDu and **G**–**I** cells were seeded in ultra-low attachment plates and spheroids were allowed to develop for 48 h. Spheroids were then treated with inhibitors targeting ATM, ATR, or DNA-Pkcs (all 0.2 µM) or DMSO as a vehicle only control for 1 h, followed by irradiation with 1 Gy PBT (or left unirradiated as a control) and growth measured by microscopy up to 15 days post-seeding. Shown is the mean spheroid volume ± S.E., analysed from three biologically independent experiments.

We then investigated the radiosensitising effect of the ATM, ATR and DNA-Pkcs inhibitors on HNSCC cells in response to PBT. A statistically significant reduction in the surviving fraction of FaDu, A253 and UMSCC6 cells was observed in the presence of either ATMi, ATRi or DNA-Pkcsi with increasing doses of PBT versus the DMSO control (Fig. 5A–C; see also Supplementary Fig. 9A–C and Supplementary Table 6). Overall, DNA-Pkcs inhibition caused the highest radiosensitising effect in all the cell lines tested (DER = 1.63–2.52), whereas the impact with ATMi was quite broad (DER = 1.16–2.70), and there was relatively less radiosensitisation with ATRi (DER = 1.34–1.76). These results were reproduced using 3D spheroid models of FaDu and A253 cells. This showed a significant reduction in spheroid growth when treated with ATMi and DNA-Pkcsi (both 0.2 µM) prior to PBT, compared to the DMSO and irradiation alone (Fig. 5D, F, G, I; see also Supplementary Fig. 10A, B and Supplementary Tables 7 and 8; DER = 2.40–3.02 for ATMi and 2.72–3.33 for DNA-Pkcsi). However, ATRi only showed a relatively mild (and non-significant) enhancement of radiosensitivity with PBT (Fig. 5E, H; see also Supplementary Fig. 10A, B and Supplementary Tables 7 and 8; DER = 1.22–1.44). A higher concentration of ATRi (1 µM) alone again proved to be relatively toxic in preventing spheroid growth, while radiosensitisation was still observable at this concentration of ATMi and DNA-Pkcsi in combination with PBT relative to the DMSO irradiated control (Supplementary Fig. 11).

**Fig. 6:**
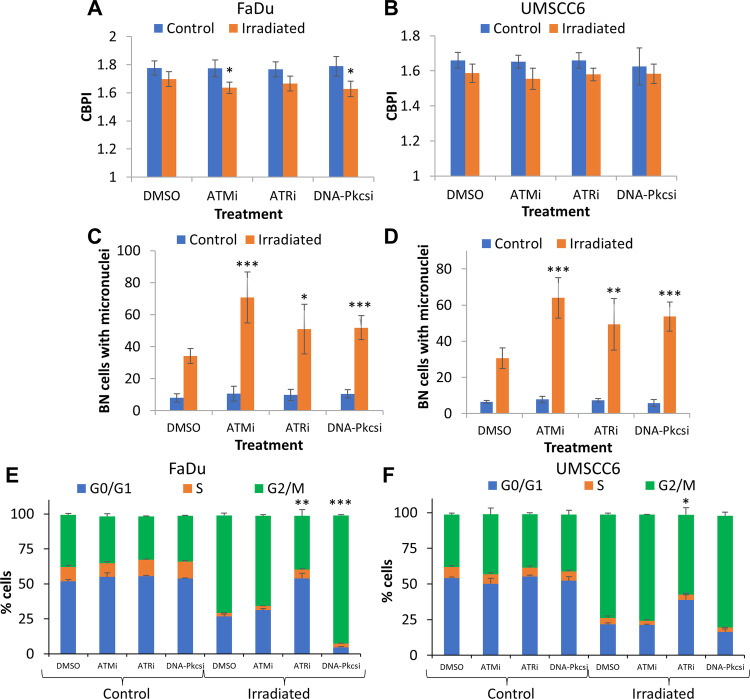
ATM, ATR and DNA-Pkcs inhibition induces chromosomal aberrations in HNSCC cells after PBT. **A** FaDu and **B** UMSCC6 cells were treated with inhibitors targeting ATM (10 nM), ATR (1 µM) and DNA-Pkcs (1 µM) or DMSO as a vehicle only control for 1 h and then treated with PBT (2 Gy). Proliferation index and micronuclei frequency was then scored by the cytokinesis-block micronuclei assay. Shown is the **A**, **B** mean CBPI ± S.E. and the **C**, **D** micronuclei frequency calculated as the mean binucleated with micronuclei scored in 1000 binucleated cells ± S.E. *p < 0.05, **p < 0.02, ***p < 0.01 as analysed by a one-sample *t* test. Alternatively following inhibitor treatment, **E** FaDu and **F** UMSCC6 cells were subject to 10 Gy X-rays and cell cycle analysis was performed 24 h post-irradiation using flow cytometry. Shown is the mean number of cells in each cell cycle phase ± S.E. *p < 0.05, **p < 0.01, ***p < 0.001 as analysed by a one-sample *t* test.

We also analysed the effect of kinase inhibition on FaDu and UMSCC6 in terms of antiproliferative ability and frequency of chromosomal abnormalities. Expectedly, CBPI was reduced in all cells following PBT, but while ATMi and DNA-Pkcsi in combination with PBT significantly reduced the proliferation index in FaDu compared to PBT only, no statistical difference was observed in UMSCC6 cells (Fig. 6A, B). ATRi with PBT had no impact on the proliferation index in both cell lines. Interestingly, micronuclei frequency was significantly elevated in the presence of either ATMi, ATRi or DNA-Pkcsi following PBT, compared to the DMSO irradiated control (Fig. 6C, D). Results on chromosome rearrangements, detected as nucleoplasmic bridges, and frequencies of nuclear buds, as a biomarker of amplified DNA elimination, are summarised (Supplementary Table 9), along with apoptotic and necrotic cell scores (Supplementary Table 10). From this analysis, there was only a significant increase in nuclear buds in UMSCC6 cells treated with DNA-Pkcsi in combination with PBT, whereas all the other end-points were non-significant relative to the DMSO control. No significant increases in necrotic and apoptotic cells were observed with the inhibitor-PBT combinations compared to PBT only. Cell cycle analysis following PBT demonstrated the expected G2/M checkpoint arrest in FaDu and UMSCC6 cells (Fig. 6E, F). PBT in combination with ATRi led to a statistically significant reduction in accumulation of G2/M cells, whereas DNA-Pkcsi after PBT showed a significant increase in G2/M phase cells compared to the DMSO irradiated control. ATMi caused no substantial differences in cell cycle phase distribution of FaDu and UMSCC6 following PBT irradiation (Fig. 6H, I).

## Discussion

Radiotherapy is key to the treatment of HNSCC, and where there is an increasing utilisation of targeted PBT that yields less damage to the normal tissues and organs at risk, and less adverse treatment side effects compared to X-rays. There is furthermore a need to identify strategies leading to the enhanced radiosensitisation of the tumour cells to overcome inherent tumour radioresistance. Given that IR, including X-ray radiation and PBT, cause the therapeutic effect through targeting and damaging the DNA particularly through the formation of potentially toxic DSBs, a logical approach is to combine inhibitors of key proteins involved in the cellular DDR to potentiate the effects of IR on tumour models. The protein kinases ATM, ATR and DNA-Pkcs are well characterised enzymes involved in the signalling and repair of DSBs, and therefore there have been some studies that have explored the effect of inhibiting these kinases in radiosensitising HNSCC [14–16]. These studies have revealed an important potential for inhibiting ATM, ATR and DNA-Pkcs in enhancing the sensitivity of HNSCC to X-ray irradiation, but which are difficult to compare due to the different inhibitors used and their ranging potency and selectivity, as well as the various cell models employed. Additionally, there is a significant lack of studies assessing the potential of DSB inhibitors to enhance the effects of PBT in HNSCC models. Here in this study, we have systematically analysed the comparative effect of potent and specific inhibitors of ATM, ATR and DNA-Pkcs on both monolayer and 3D spheroid models of HNSCC in response to X-ray radiation and PBT for direct analysis.

We observed that inhibitors targeting ATM, ATR or DNA-Pkcs can decrease the clonogenic survival of several HNSCC cells in response to both X-rays but also PBT. DNA-Pkcsi appeared particularly effective in reducing clonogenic survival of all cell lines in combination with X-rays (DER = 1.41–2.85) and which was comparable to that observed with PBT (DER = 1.63-2.52). However, ATRi had an apparent less radiosensitising effect (DER = 1.15–2.49 and DER = 1.34–1.76 with X-rays and PBT, respectively). This difference in the radiosensitising potential of the inhibitors was more evident using 3D spheroids, where DNA-Pkcsi (but also ATMi) caused significantly reduced growth with both X-rays and PBT, whereas ATRi was less effective as a radiosensitiser but showed toxic affects as a monotherapy. It should be noted here that experiments with spheroids were performed with the inhibitors continually present throughout the analysis period post-irradiation (albeit at half the dose initially applied), whereas with clonogenic survival assays the cell media is entirely removed and replaced with drug-free media post-irradiation. Nevertheless, these findings are in keeping with our previous study using alternative and less potent kinase inhibitors [22], and which ultimately support that inhibition of DNA-Pkcs but also ATM are able to potentiate the cell killing effects of both X-rays and PBT. DNA-Pkcs inhibitors, such as IC87361 and KU-57788, have previously been shown to enhance the sensitivity of HNSCC cell lines after X-ray irradiation [18] and PBT [22], respectively. These data correlate with our findings presented here using a more potent inhibitor of DNA-Pkcs, AZD7648. Similarly, the ATM inhibitor GSK635416A has been tested on several HNSCC cell lines (UT-SCC2, UT-SCC8, UT-SCC24A, UT-SCC36 and UT-SCC40) following X-ray irradiation, and was proven to reduce cell survival compared to a vehicle only control [19]. While ATM inhibition through KU-55933 caused enhanced radiosensitivity in both 2D and 3D spheroid models of HNSCC following X-rays and protons [22].

Interestingly, several studies have demonstrated how different ATR inhibitors can improve the radiosensitisation of HNSCC cells in response to X-ray irradiation [17, 20–22, 23]. While our data, particularly from clonogenic survival assays, do not disagree with this evidence, using spheroid models suggests that ATRi alone is potent as a monotherapy and which must be considered in the further evaluation of this as a radiosensitiser. We also observed significant toxicity of the inhibitor alone at higher concentrations using clonogenic assays, Similar observations have been seen in a previous study, where inhibition of ATR via AZD6738 appeared to display only a mild enhancement of X-ray irradiation in preventing spheroid growth of FaDu cells due to the potency of the drug alone [21].

In terms of mechanism of action, we confirmed that inhibition of ATM, ATR and DNA-Pkcs lead to delays in DSB repair, as expected. These inhibitors also induced a significant increase in micronuclei formation following both X-ray and PBT compared to the DMSO irradiated control cells. This correlates with the intense micronucleation previously found in irradiated cancer cells following ATM inhibition with AZD1390, M3541 and M4076 [24, 25], or through DNA-Pkcs inhibition with AZD7648 and M3814 [26, 27]. ATR inhibition (AZD6738) has been previously investigated in Cal27 and FaDu HNSCC cells post-irradiation, where the majority of micronuclei were acentric chromosomal fragments lacking a centromere [21]. This confirms our observation regarding the aneuploidogenic capacity of the DDR inhibitor and more specifically the absence of centromere signal in the in situ hybridisation experiment in ATR-depleted cells. Interestingly, DNA-Pkcs-depleted HNSCC cells showed reduced proliferation activity compared to DMSO controls after both X-ray and PBT, which could be explained by the potential accelerated senescence induction in targeted DNA-Pkcs irradiated cells [28–30], coupled with abrogated radiation-induced DSB repair, confirmed by our comet assay findings.

Taken together, our findings provide further support that inhibiting the key proteins involved in DNA DSB repair, particularly via ATM and DNA-Pkcs, can enhance the radiosensitivity of HNSCC cells in response to X-rays and PBT. This suggests that NHEJ, coordinated by ATM and DNA-Pkcs, could be the primary DSB repair pathway employed following both radiation types and which is supported by other evidence [31, 32]. However, it is important to state that the X-rays and PBT used in this study are both low LET, whereas there are suggestions that high-LET radiation shows a great reliance on the HR pathway (ATR-dependent) in repairing complex DNA damage, including complex DSBs [3]. Therefore, our future work will investigate the effect of ATM, ATR and DNA-Pkcs depletion in HNSCC cells treated with radiation of increasing LET. Additionally, we aim to further explore the potential for the kinase inhibitors in radiosensitising more complex models of HNSCC, including patient-derived organoids and in vivo models, with a view to defining and optimising treatment strategies that could be translated to the clinic for patient benefit.

## Materials and methods

### Antibodies and inhibitors

The following antibodies were used: ATM, ATR, phosphorylated ATM and phosphorylated DNA-Pkcs (ab78, ab2905, ab81292 and ab18192, respectively; Abcam, Cambridge, UK), phosphorylated ATR (58014; Cell Signalling Technology, Massachusetts, USA), DNA-Pkcs (sc-9051; Santa Cruz Biotechnology, Heidelberg, Germany), and tubulin (T6199; Sigma-Aldrich, Gillingham, UK). Goat anti-mouse Alexa Fluor 680 or goat anti-rabbit Alexa Fluor 800 secondary antibodies for immunoblotting were from Life Technologies (Paisley, UK). Inhibitors for ATM (AZD1390; ATMi), ATR (AZD6738; ATRi) and DNA-Pkcs (AZD7648; DNA-Pkcsi) were kindly provided by AstraZeneca UK.

### Cell culture and irradiation sources

Oropharyngeal (UMSCC6 and UMSCC74A) and laryngeal (UMSCC12) squamous cell carcinoma cells were kindly provided by Professor T. Carey, University of Michigan, USA. Hypopharyngeal squamous cell carcinoma cells (FaDu), submaxillary salivary gland carcinoma cells (A253) and pharyngeal carcinoma cells (Detroit 562) were purchased from ATCC (Teddington, UK). All cell lines were authenticated in our laboratory by STR profiling, and were routinely cultured as monolayers in 5% CO_2_ at 37 °C as previously described [22]. Cells were cultured in Dulbecco’s Modified Eagle Medium (DMEM) supplemented with 10% fetal bovine serum, 2 mM L-glutamine, 1× penicillin–streptomycin and 1× non-essential amino acids, except for FaDu cells and Detroit 562 cells which were cultured in Modified Eagle Medium (MEM). For cell irradiations, cells were exposed to either X-rays (150 kV; CellRad X-ray irradiator, Faxitron Bioptics, Tucson, USA), or with a horizontal, passive-scattered proton beam line of 28 MeV maximal energy delivered by the MC-40 cyclotron (dose rates of ~5 Gy/min).

### Clonogenic assays

A defined number of cells were seeded in triplicate into 6-well plates (X-rays), or into 35 mm dishes (PBT), and allowed to attach for ~16 h. Cells were treated with ATMi (10 nM), ATRi (1 µM) or DNA-Pkcsi (1 µM) for 1 h prior to irradiation. Following treatment with X-rays or PBT, the media was removed and replaced with fresh media. Plating efficiencies for the cells were as follows: FaDu, A253 and UM-SCC12 (~20%), UMSCC6, UMSCC74A and Detroit 562 (~15%). An increasing number of cells were plated for increasing IR doses to allow for plating efficiencies. Colonies were allowed to grow for 7–14 days, prior to fixing and staining with 6% glutaraldehyde and 0.5% crystal violet for 30 min. Plates/dishes were washed, left to air dry overnight and colonies were counted using the GelCount colony analyser (Oxford Optronics, Oxford, UK). Colony counting settings were optimised for all cell lines, based on inclusion of distinct colonies of specific size and intensity, although the same settings were used across the various treatments. Relative colony formation (surviving fraction) was expressed as colonies per treatment level versus colonies that appeared in the untreated control. Results were accumulated from at least three independent biological experiments.

### Spheroid growth assay

Spheroid assays were performed as previously described [22]. In brief, cells were seeded at 500–1000 cells/well in triplicate in 100 µl Advanced MEM (Gibco Life Technologies) containing 1% B27 supplement, 0.5% N2 supplement, 2 mM L-glutamine, 1× penicillin–streptomycin, 5 µg/ml heparin, 20 ng/µl epidermal growth factor (EGF) and 10 ng/µl fibroblast growth factor (FGF) in 96-well ultra-low attachment plates (Corning B.V. Life Sciences, Amsterdam, The Netherlands). After 48 h, at which the spheroids were ~200 µm in size, and where appropriate, the inhibitors were added to the spheroids at a concentration of 0.2 or 1 µM for 1 h, and then irradiated. Immediately following irradiation, 50 µl culture media was removed and replaced by 50 µl fresh media (without inhibitor), meaning that the spheroids were cultured in inhibitor-containing media throughout the culture period but at half the original dose. The growth of spheroids was monitored up to 15 days post-seeding by image capture using the EVOS M5000 Imaging System (Life Technologies, Paisley, UK). The diameter (*d*) of the spheroids was measured by using ImageJ and was converted into spheroid volume (*V*) by using the formula *V* = 4/3 × *π*(*d*/2).

### Neutral comet assay

Cells were trypsinised, diluted to 1 × 10^5^ cells/ml and 250 µl aliquots of the cell suspension placed into the wells of a 24-well plate which was placed on ice. Cells were irradiated (4 Gy) and embedded on a microscope slide in low melting agarose (Bio-Rad, Hemel Hemp-stead, UK). The slides were incubated for up to 4 h at 37 °C in a humidified chamber to allow for DNA repair, prior to cell lysis in buffer containing 2.5 M NaCl, 100 mM EDTA, 10 mM Tris-HCl pH 10.5, 1% N-lauroylsarcosine, 1% DMSO and 1% (v/v) Triton X-100. Slides were washed three times with cold electrophoresis buffer (1× TBE buffer (pH 8.3)), then placed in cold electrophoresis buffer (1× TBE buffer (pH 8.3)) in the dark for 25 min to allow the DNA to unwind, prior to electrophoresis at 25 V, ~20 mA for 25 min. Slides were washed three times with 1× PBS before allowing to dry overnight. Slides were rehydrated for 30 min in water (pH 8.0), stained for 30 min with SYBR Gold (Life Technologies, Paisley, UK) diluted 1:10,000 in water (pH 8.0) and again dried overnight. Cells (50 per slide, in duplicate) were analysed from the dried slides using the Komet 6.0 image analysis software (Andor Technology, Belfast, Northern Ireland) and % tail DNA values averaged from at least three independent biological experiments.

### Cytokinesis-block micronucleus assay

The assessment of genotoxicity on HNSCC cells was performed as previously reported [33]. Briefly, 24 h after seeding, the cells were exposed to ATMi (10 nM), ATRi (1 µM) or DNA-Pkcsi (1 µM) for 1 h, then irradiated with X-rays or PBT (2 Gy). Cytochalasin B (5 µg/ml, Sigma-Aldrich, Gillingham, UK) was added to block the first cytokinesis process at a specific cell line-dependent timepoint (for UMSCC74A, UMSCC6 and FaDu at 15, 20 and 18 h, respectively). Cells were harvested after 24 h, then treated with a hypotonic solution (75 mM KCl) for 3 min, prefixed in 3:5 methanol:acetic acid, washed once with methanol and subsequently fixed twice with a 6:1 methanol:acetic acid. Finally, the cell solution was dropped onto cold glass microscope slides. Staining was performed by immersing the air-dried slides in a 2% Giemsa solution in Sorensen’s buffer (pH 6.8). Binucleated cells (2000) were examined for each experimental point in a blind mode (1000 from each independent culture replicate) using a Nikon Eclipse 800 optical microscope (final magnification of ×400). The scoring criteria adopted by Fenech [34] was followed for each endpoint. Briefly, we evaluated the frequency of binucleate micronucleated cells relative to binucleate cells containing one or more micronuclei per 1000 binucleated cells. Moreover, 500 cells were scored to evaluate the percentage of mononucleated, binucleated and multinucleated cells, and the cytokinesis-block proliferation index (CBPI) was calculated as an index of cytotoxicity by comparing values in the treated and control cultures. The CBPI indicates the average number of cell cycles per cell during the period of exposure to Cytochalasin B, and may be used to calculate cell proliferation. Finally, other damage events were scored in once-divided binucleated cells per 1000 cells, namely: (i) nucleoplasmic bridges, a biomarker of DNA misrepair and/or telomere end-fusions and (ii) nuclear buds (NBUDs), a biomarker of elimination of amplified DNA and/or DNA repair complexes. The number of apoptotic and necrotic cells were also evaluated.

### Immunoblotting and cell cycle analysis

Whole cell extracts were prepared, separated by SDS-PAGE electrophoresis and analysed by quantitative immunoblotting using the Odyssey image analysis system (Li-cor Biosciences, Cambridge, UK), as previously described [35]. For cell cycle analysis, cells were trypsinised, washed twice with ice cold PBS (100 × *g* for 5 min at 4 °C), fixed with ice cold 70% ethanol and kept at 4 °C until analysis, as described previously [36].

### Statistical analysis

All experiments were performed in at least triplicate as separate independent, biological experiments. Statistical analysis of cytokinesis-block micronucleus assay results was performed using a one-way analysis of variance (ANOVA). Dose enhancement ratios were calculated from spheroid growth assays by comparing the inhibition of growth achieved over a period up to 15 days post-seeding in the inhibitor-treated cells versus the DMSO-treated cells, both in the presence of irradiation (1 Gy dose). Statistical analysis of the spheroid growth assays was performed using a one-way ANOVA. Statistical analysis of clonogenic survival data was performed using the CFAssay for R package [37], which uses the linear-quadratic model (LQ model) to compare different treatment responses across increasing radiation doses. Dose enhancement ratios were calculated from linear quadratic fitting at a surviving fraction of 0.5, comparing the doses required to achieve the same survival in the DMSO- versus to the inhibitor-treated cells. Statistical analysis of DNA DSB damage quantified through neutral comet assays and the flow cytometry data was performed using either a one- or two-sample *t* test.

### Supplementary information

Supplementary Data

## Data Availability

Any source or other data will be made available from the corresponding author upon reasonable request.
