# Supplementary material for: Inhibition of key DNA double strand break repair protein kinases enhances radiosensitivity of head and neck cancer cells to X-ray and proton irradiation
Source: Cell Death Discov. 2024 Jun 12;10:282. doi: 10.1038/s41420-024-02059-3 (PMC11169544; doi:10.1038/s41420-024-02059-3)
Supplement: Supplementary file 1 — Supplementary Data [file 41420_2024_2059_MOESM1_ESM.docx]

**SUPPLEMENTARY DATA**

**Supplementary Table 1. Statistical analysis of clonogenic survival assay data of HNSCC cells treated with DSB protein kinases inhibitors and X-ray radiation**.


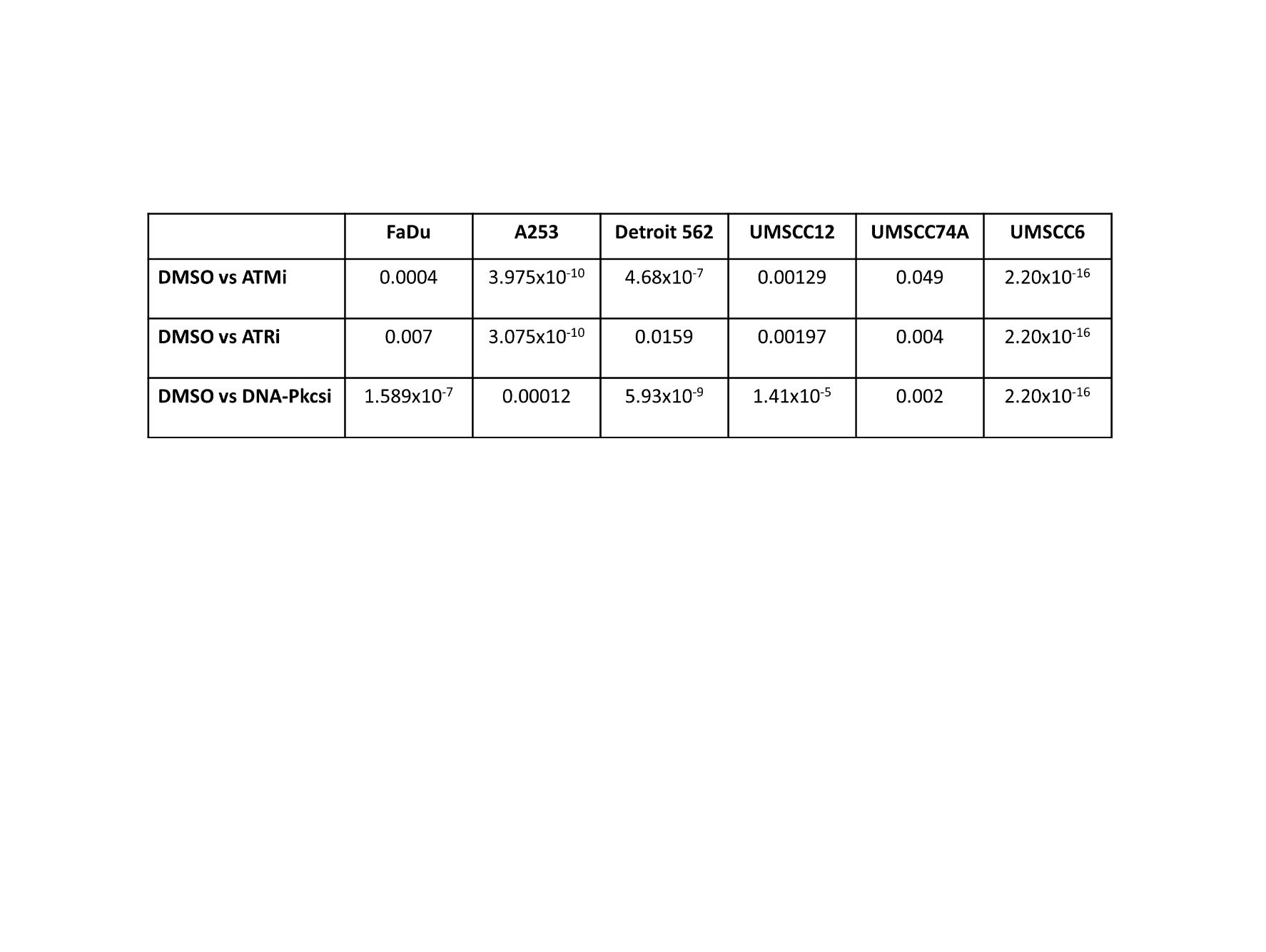


**Supplementary Table 2. Dose enhancement ratios derived from 3D spheroid growth assays of HNSCC cells treated with DSB protein kinases inhibitors and X-ray radiation**.


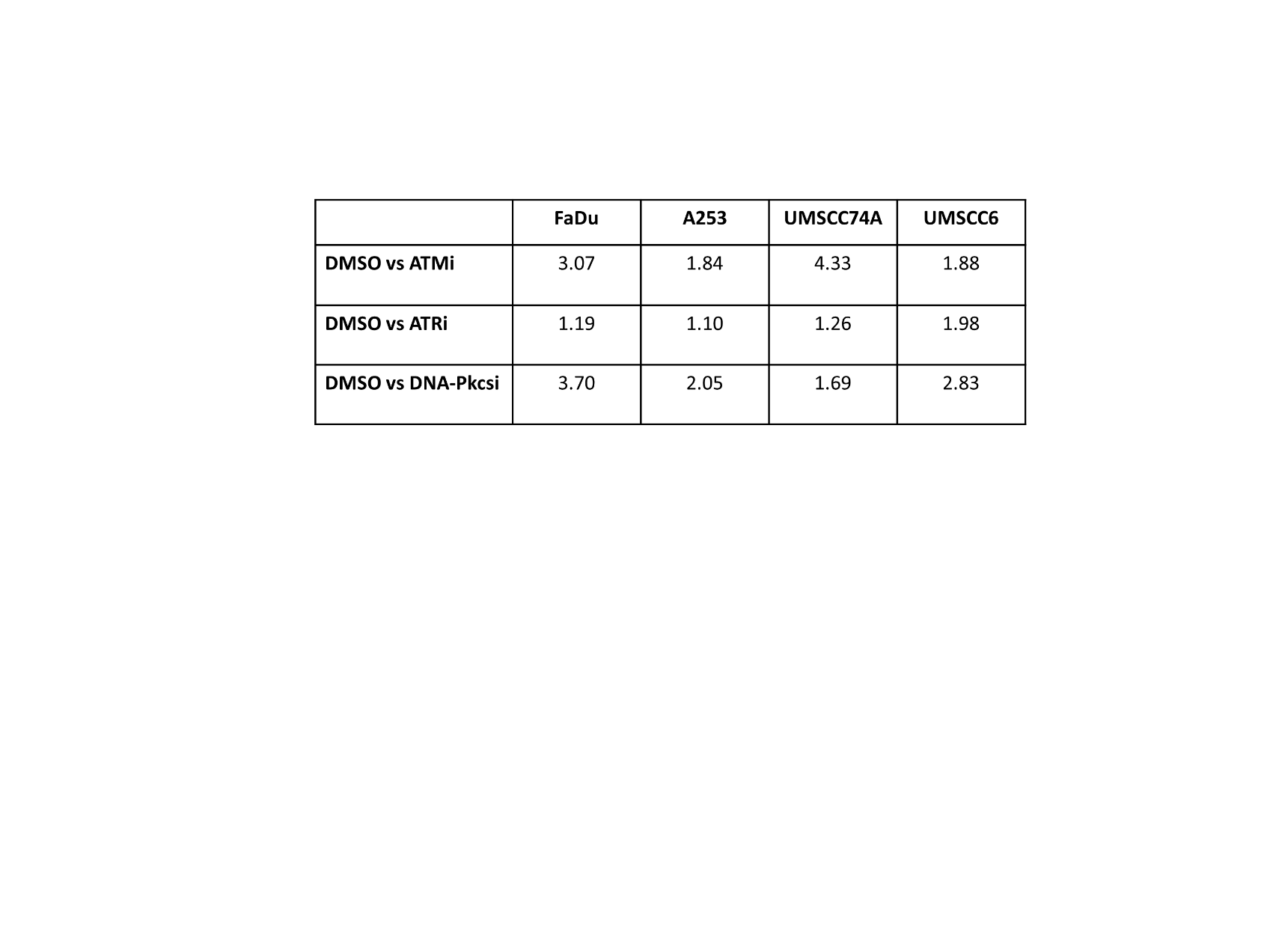


**Supplementary Table 3. Statistical analysis of 3D spheroid growth assay data of HNSCC cells treated with DSB protein kinases inhibitors and X-ray radiation**.

**
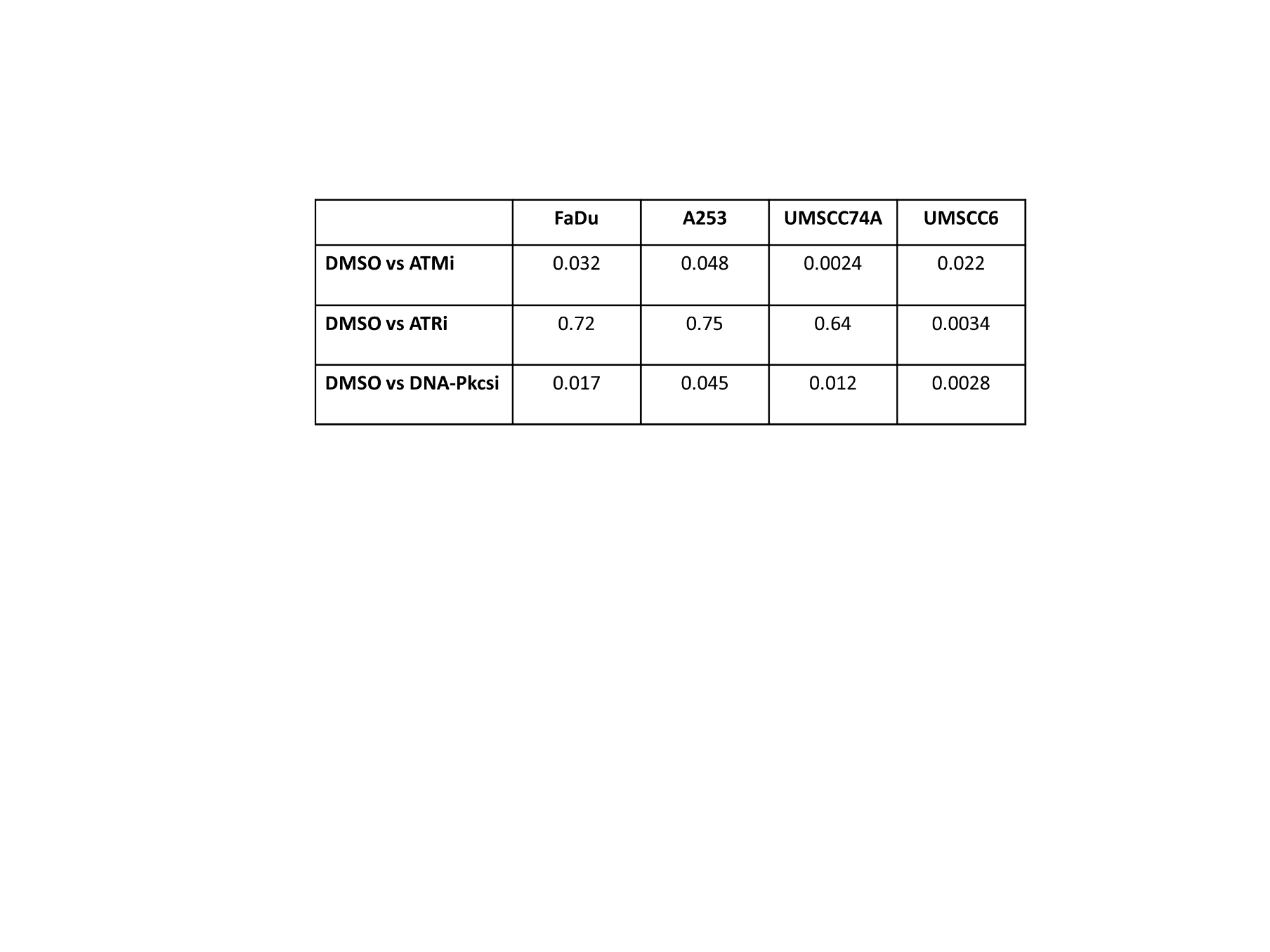
**

**Supplementary Table 4. Analysis of chromosomal aberrations of HNSCC cells treated with DSB protein kinases inhibitors and X-ray irradiation**.


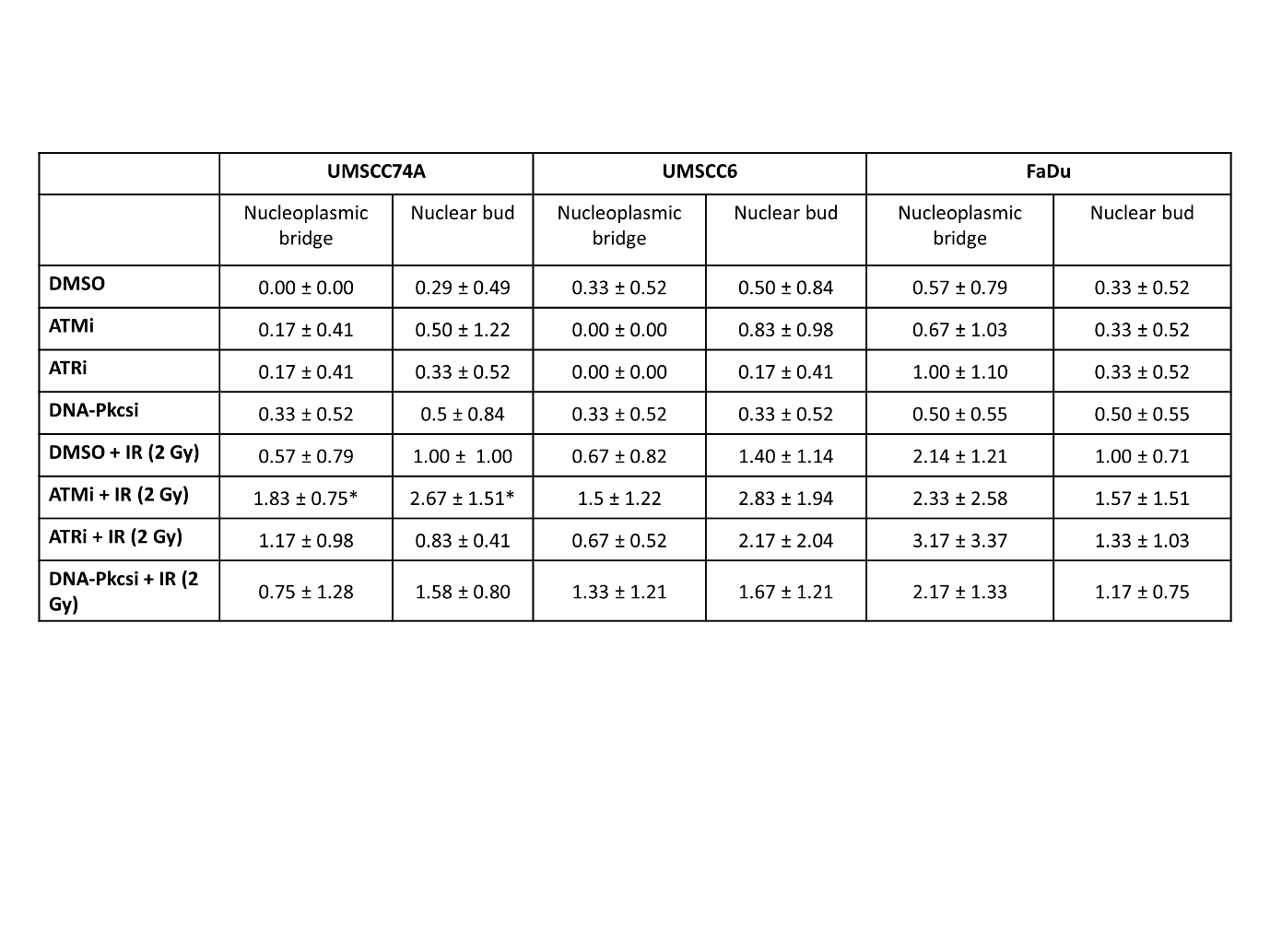


*p<0.05 as analysed by a one sample *t*-test comparing the drug plus irradiation sample versus the DMSO irradiated control.

**Supplementary Table 5. Analysis of cell death of HNSCC cells treated with DSB protein kinases inhibitors and X-ray irradiation**.


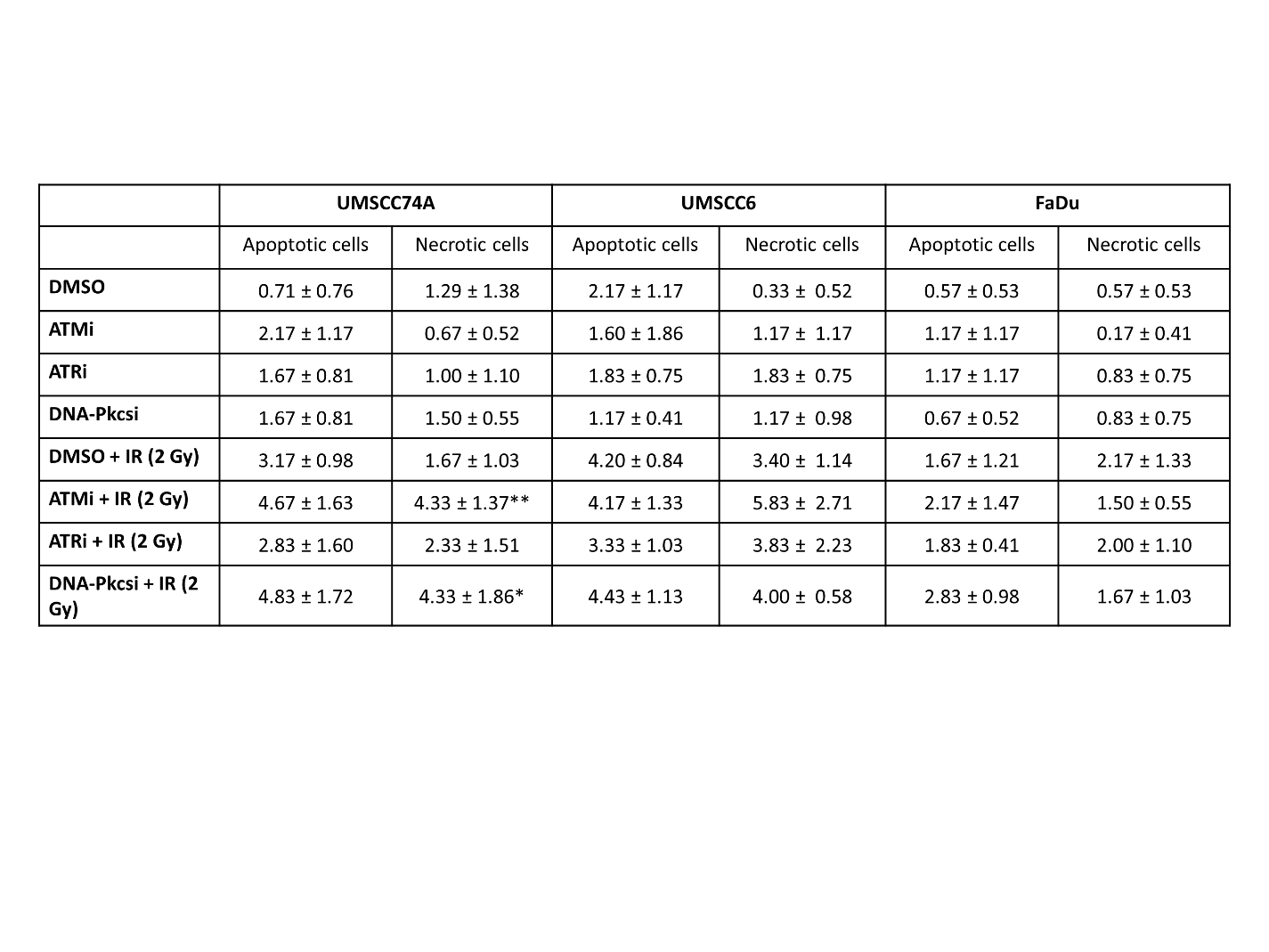


*p<0.05, **p<0.01 as analysed by a one sample *t*-test comparing the drug plus irradiation sample versus the DMSO irradiated control.

**Supplementary Table 6. Statistical analysis of clonogenic survival assay data of HNSCC cells treated with DSB protein kinases inhibitors and PBT**.


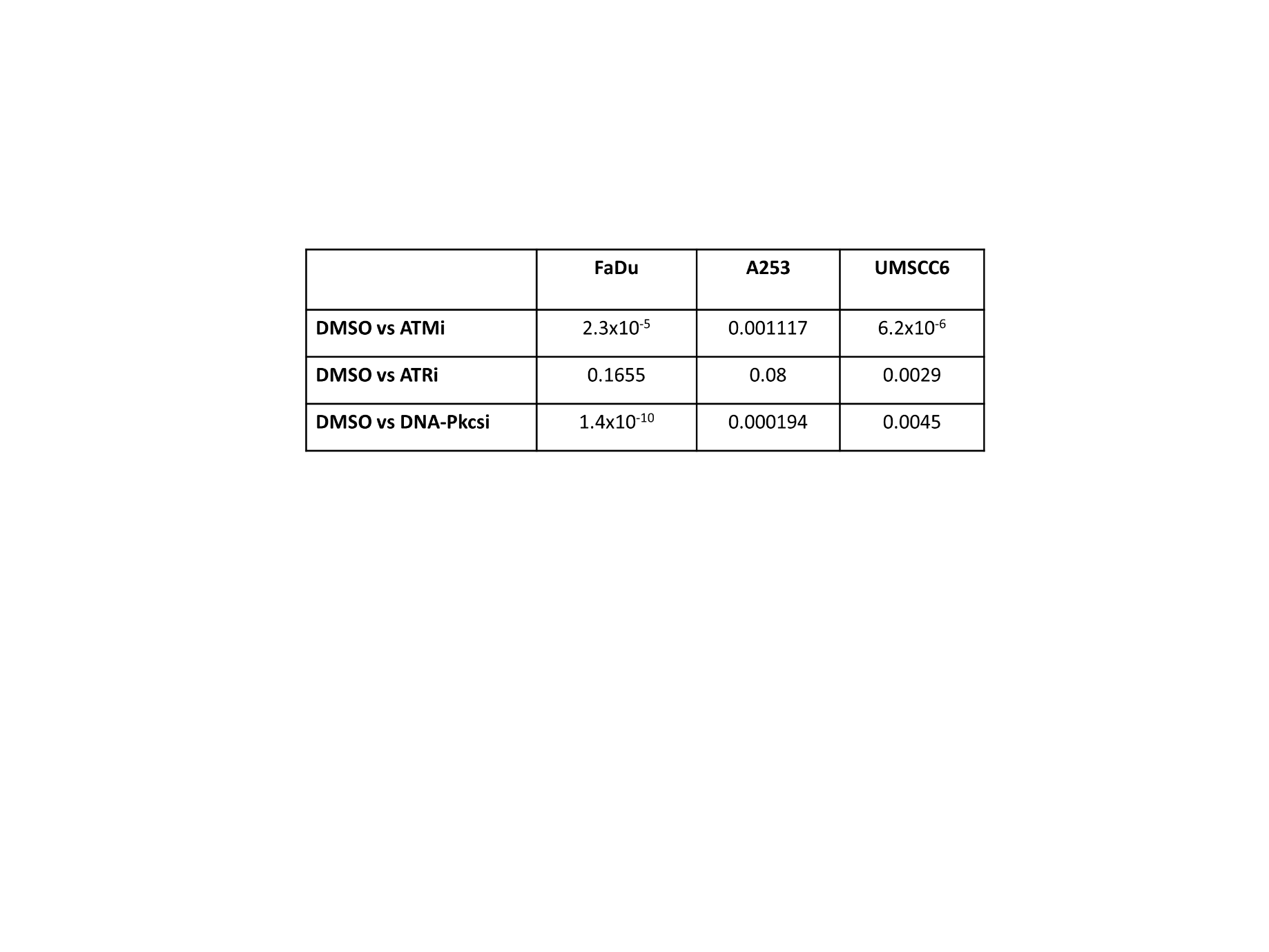


**Supplementary Table 7. Dose enhancement ratios derived from 3D spheroid growth assays of HNSCC cells treated with DSB protein kinases inhibitors and PBT**.


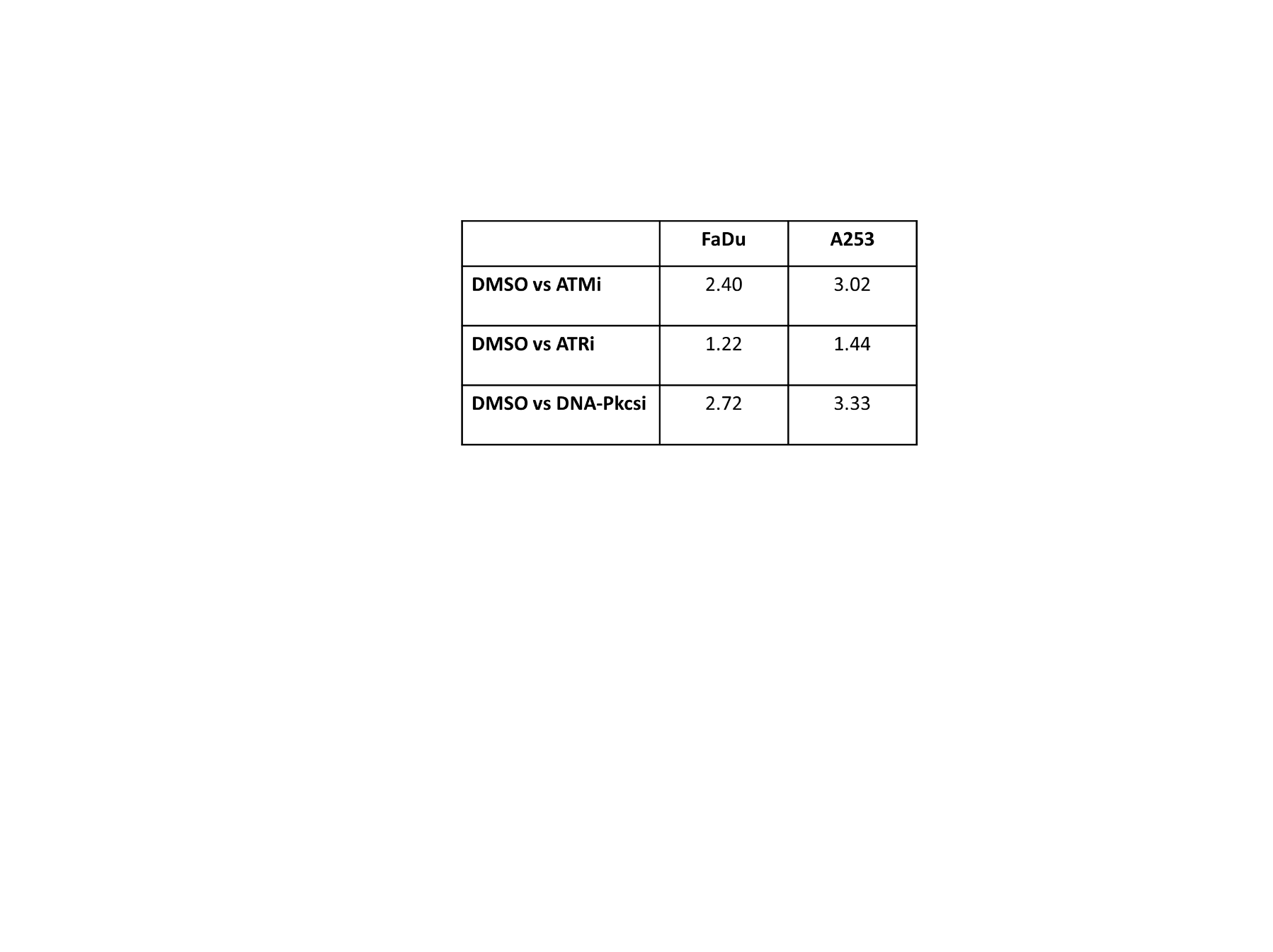


**Supplementary Table 8. Statistical analysis of 3D spheroid growth assay data of HNSCC cells treated with DSB protein kinases inhibitors and PBT**.

**
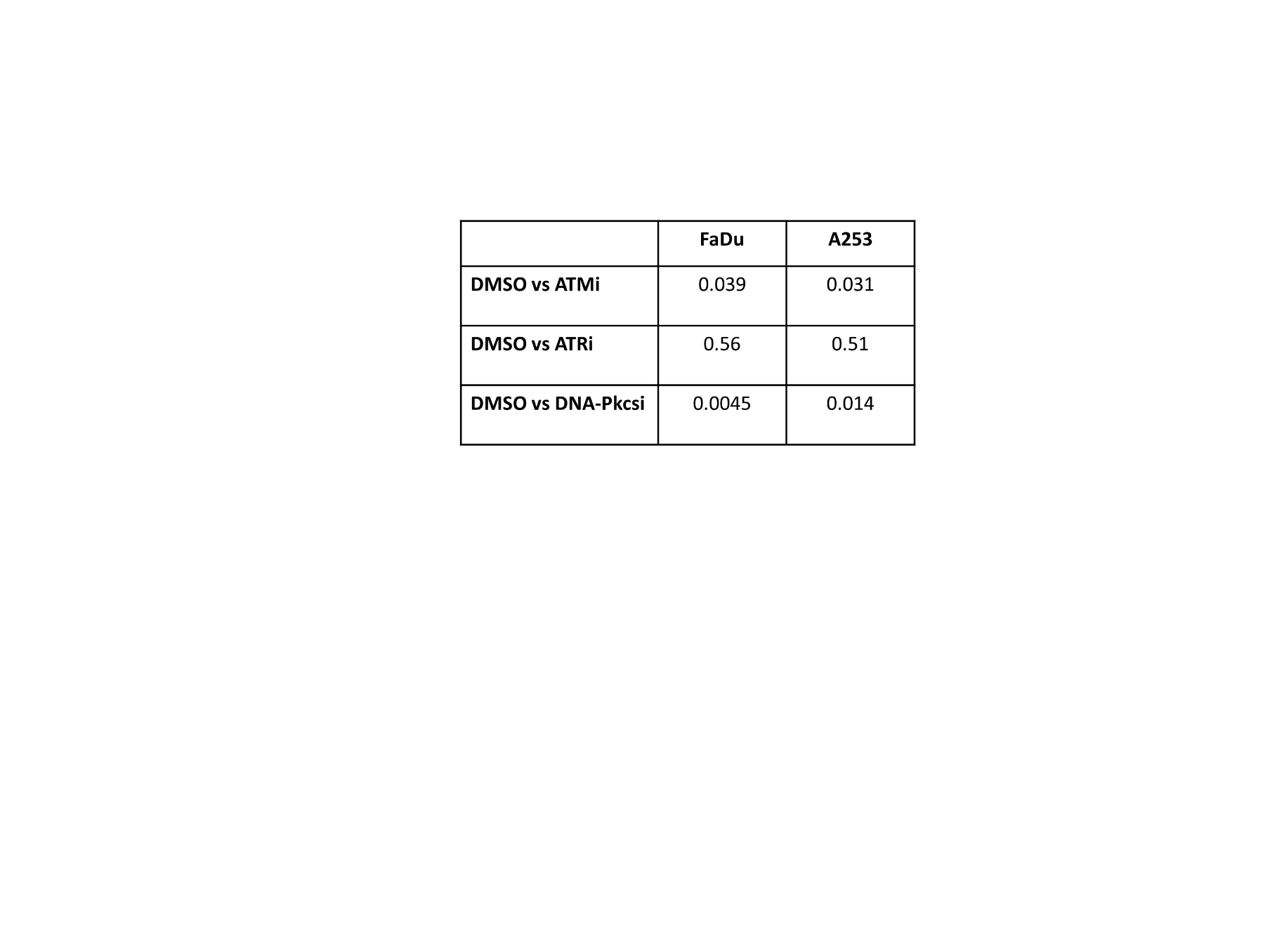
**

**Supplementary Table 9. Analysis of chromosomal aberrations of HNSCC cells treated with DSB protein kinases inhibitors and PBT**.


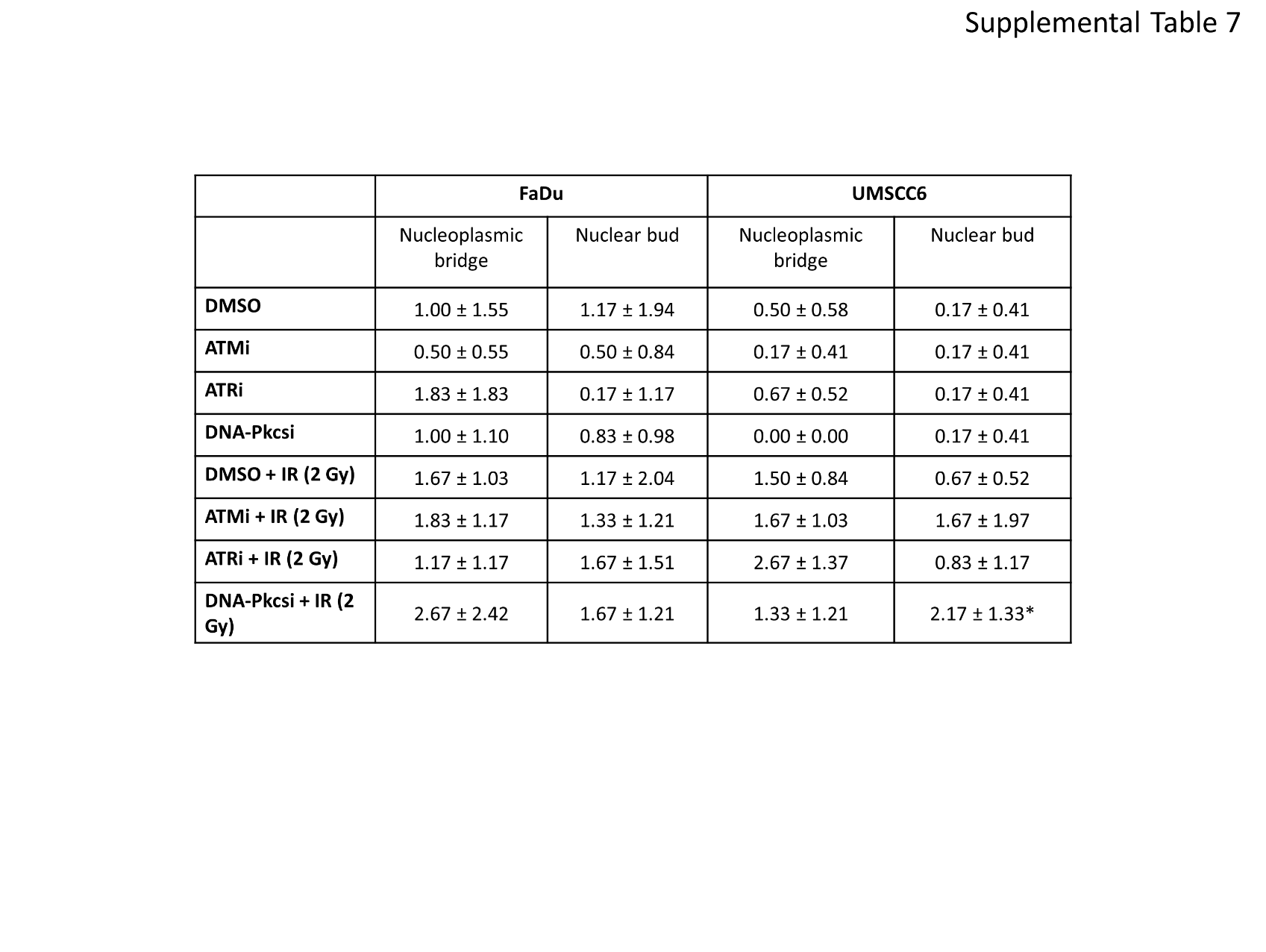


*p<0.05 as analysed by a one sample *t*-test comparing the drug plus irradiation sample versus the DMSO irradiated control.

**Supplementary Table 10. Analysis of cell death of HNSCC cells treated with DSB protein kinases inhibitors and PBT**.


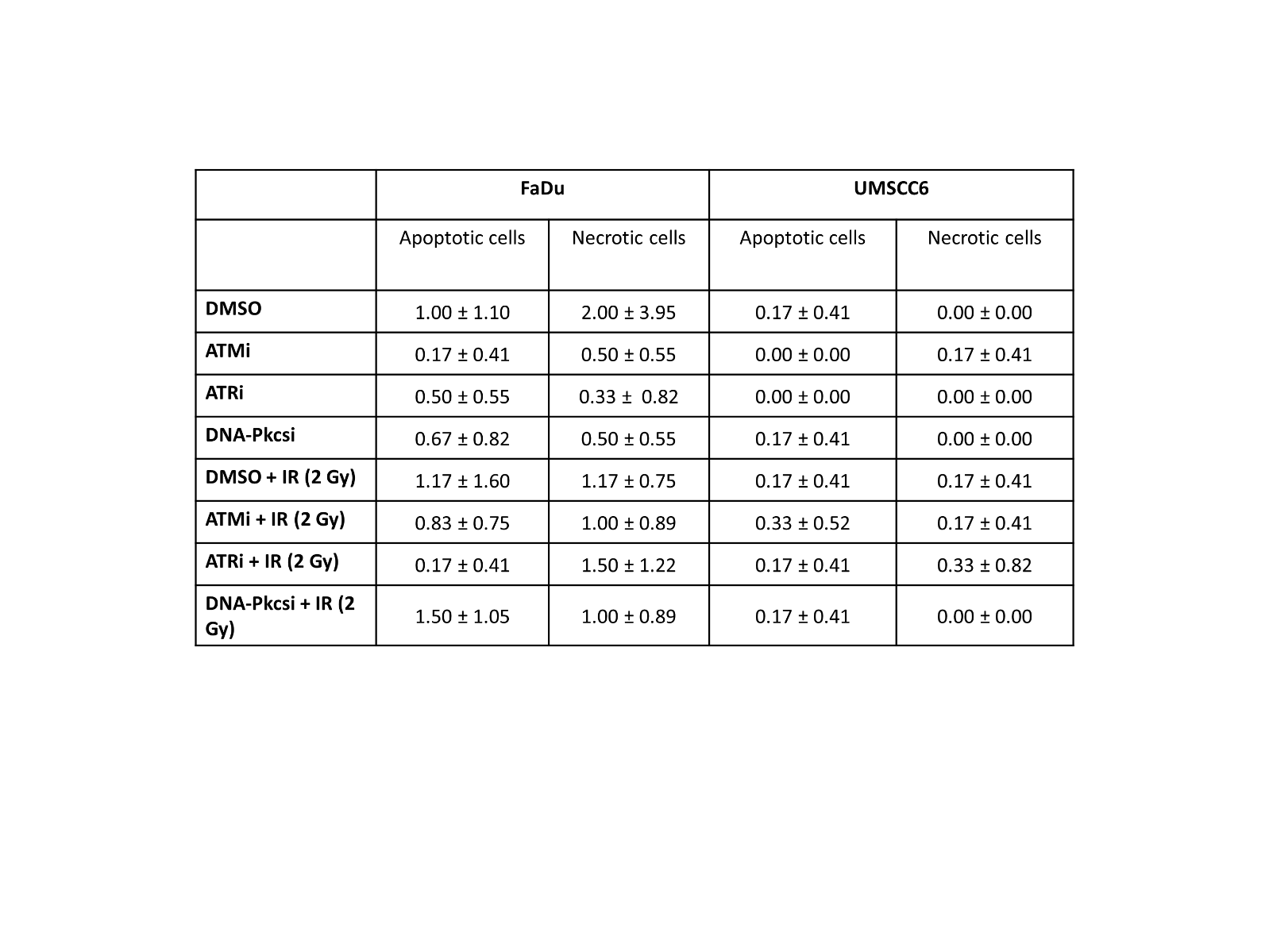


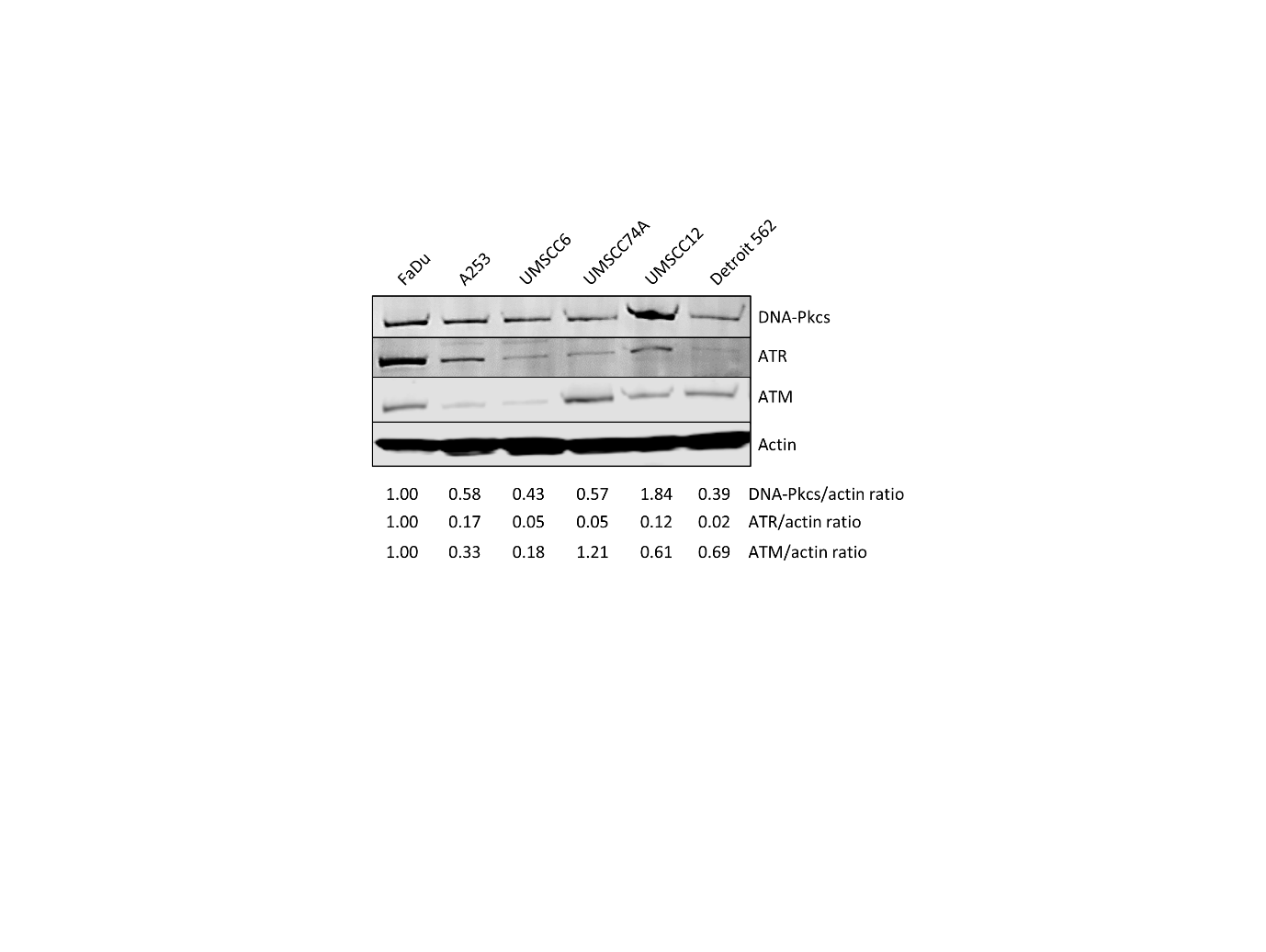


**Supplementary Figure 1. Levels of DSB protein kinases in HNSCC cells**. Whole cell extracts were prepared from the HPV-negative HNSCC cells indicated, and analysed by immunoblotting with antibodies against ATM, ATR or DNA-Pkcs, and using actin as a loading control. Representative images are shown, along with the relative ratio of proteins to actin, normalised to those in FaDu cells which was set to 1.0.


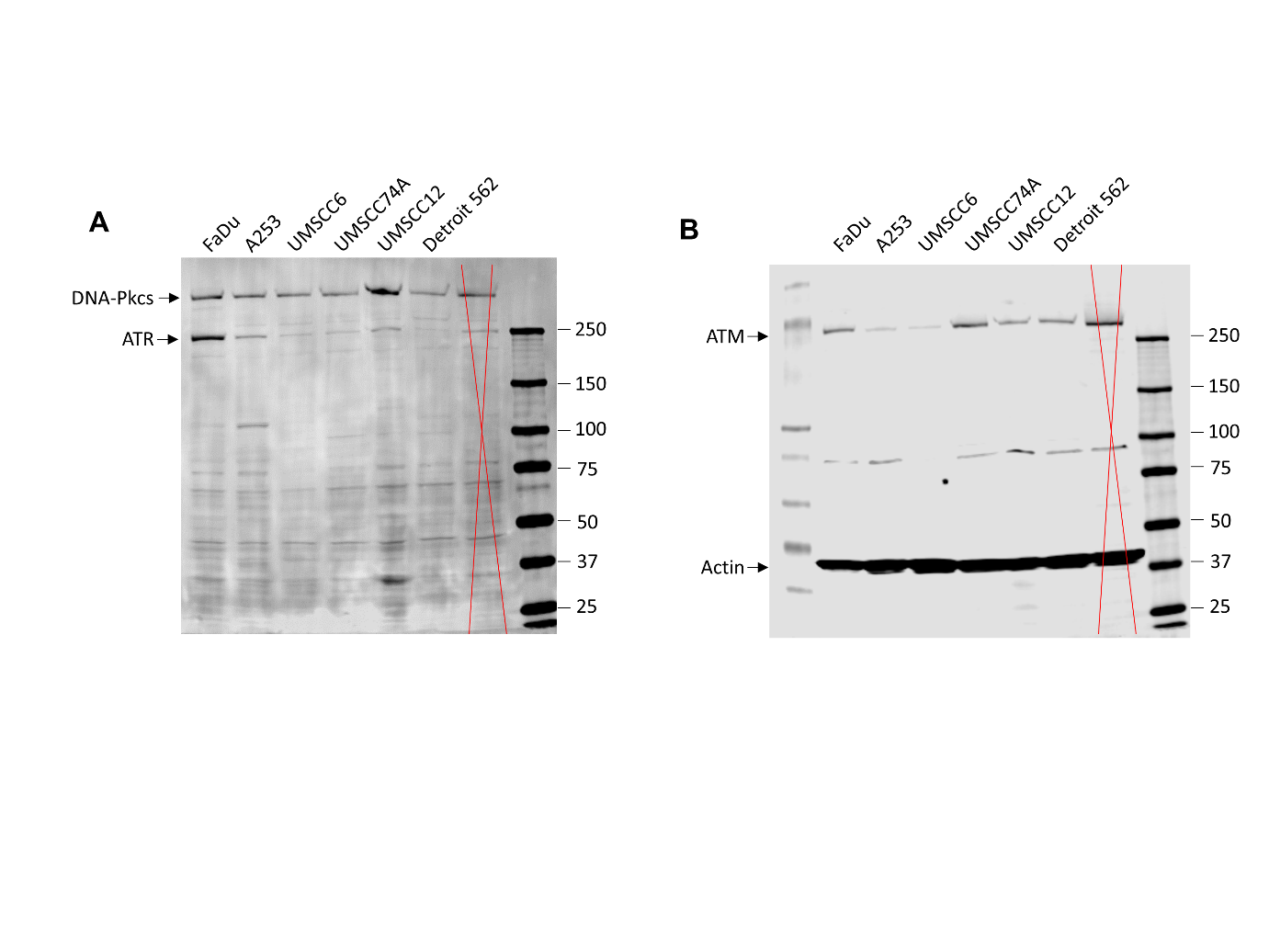


**Supplementary Figure 2. Full length blots of Supplementary Figure 1.**


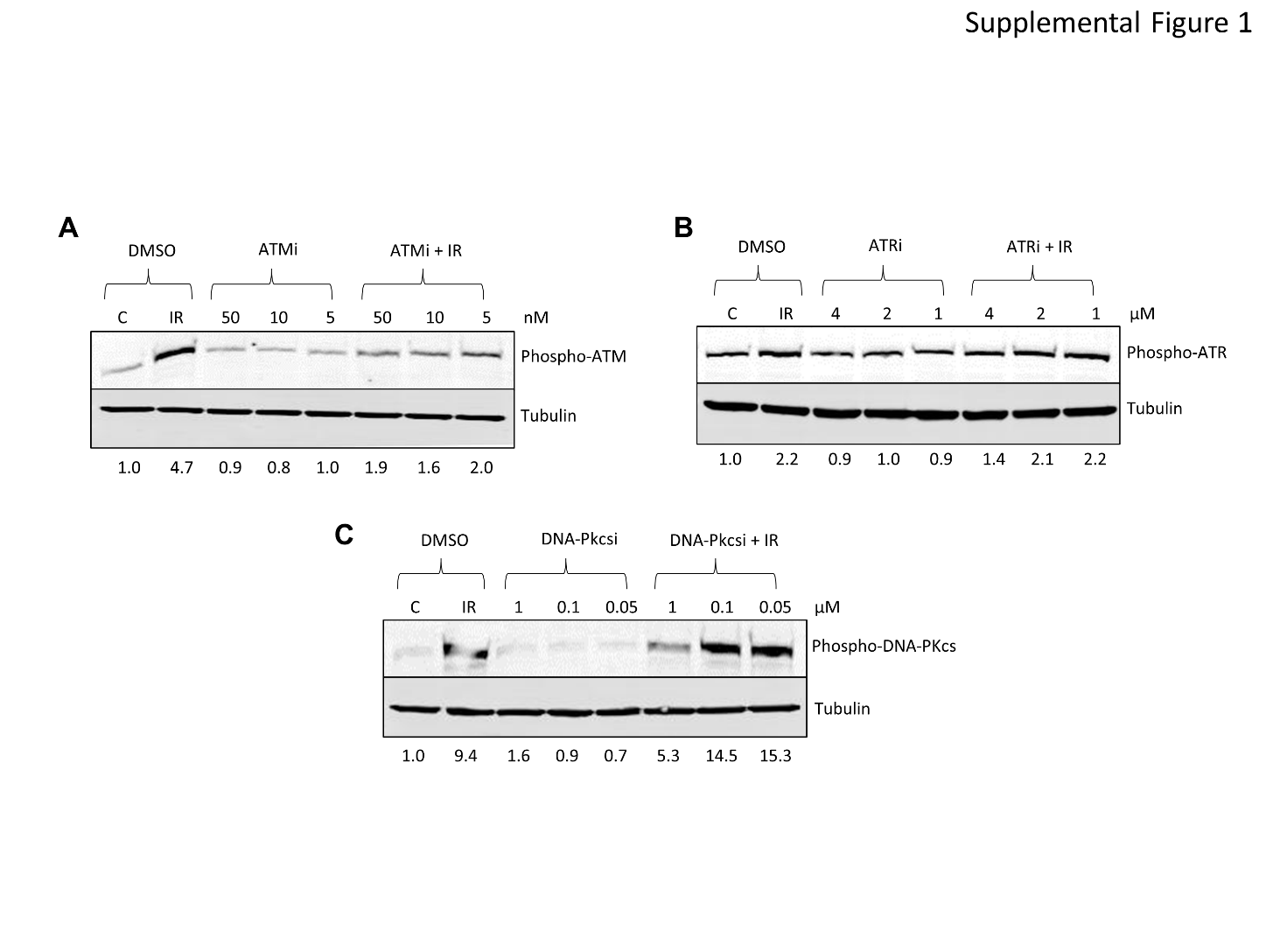


**Supplementary Figure 3. Effective inhibition of DSB protein kinases in response to X-ray irradiation**. HPV-negative (UMSCC74A) HNSCC cells were preincubated with inhibitors targeting ATM (5-50 nM), ATR (1-4 µM) or DNA-Pkcs (0.05-1 µM) for 1 h, then either unirradiated (indicated as C) or irradiated (IR) with 4 Gy X-rays. Cells were harvested at either 4 h (ATM, DNA-Pkcs) or 8 h (ATR) post-irradiation. Whole cell extracts were prepared and analysed by immunoblotting with site-specific antibodies against (A) ATM phosphorylated on serine 1981 (pATM), (B) ATR phosphorylated on threonine 1989 (pATR) or (C) DNA-Pkcs phosphorylated on serine 2056 (pDNA-Pkcs), and using tubulin as a loading control. Representative images are shown, along with the relative ratio of phosphorylated proteins to tubulin, normalised to those in the unirradiated DMSO-treated cells which was set to 1.0.


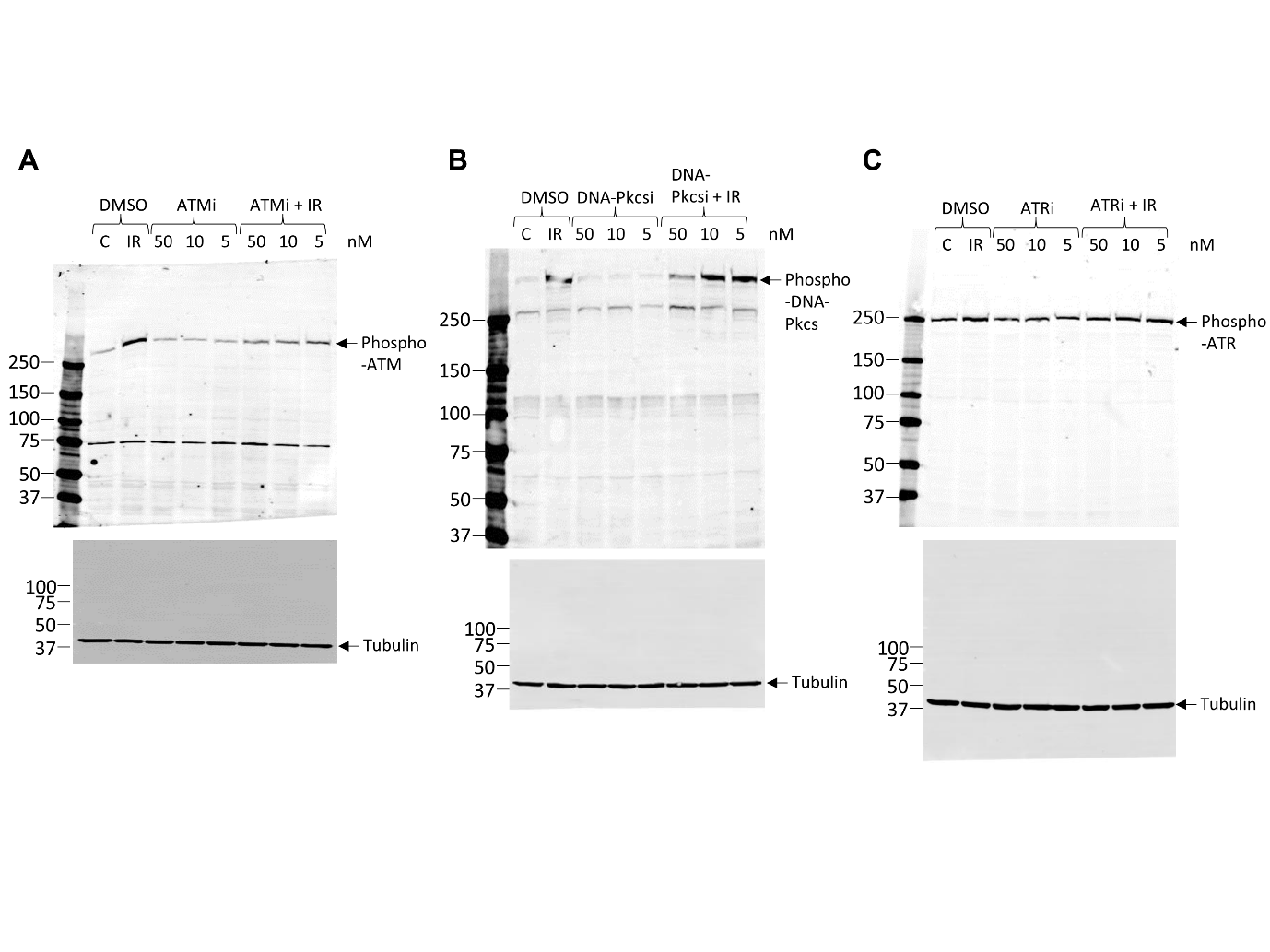


**Supplementary Figure 4. Full length blots of Supplementary Figure 3.**

**
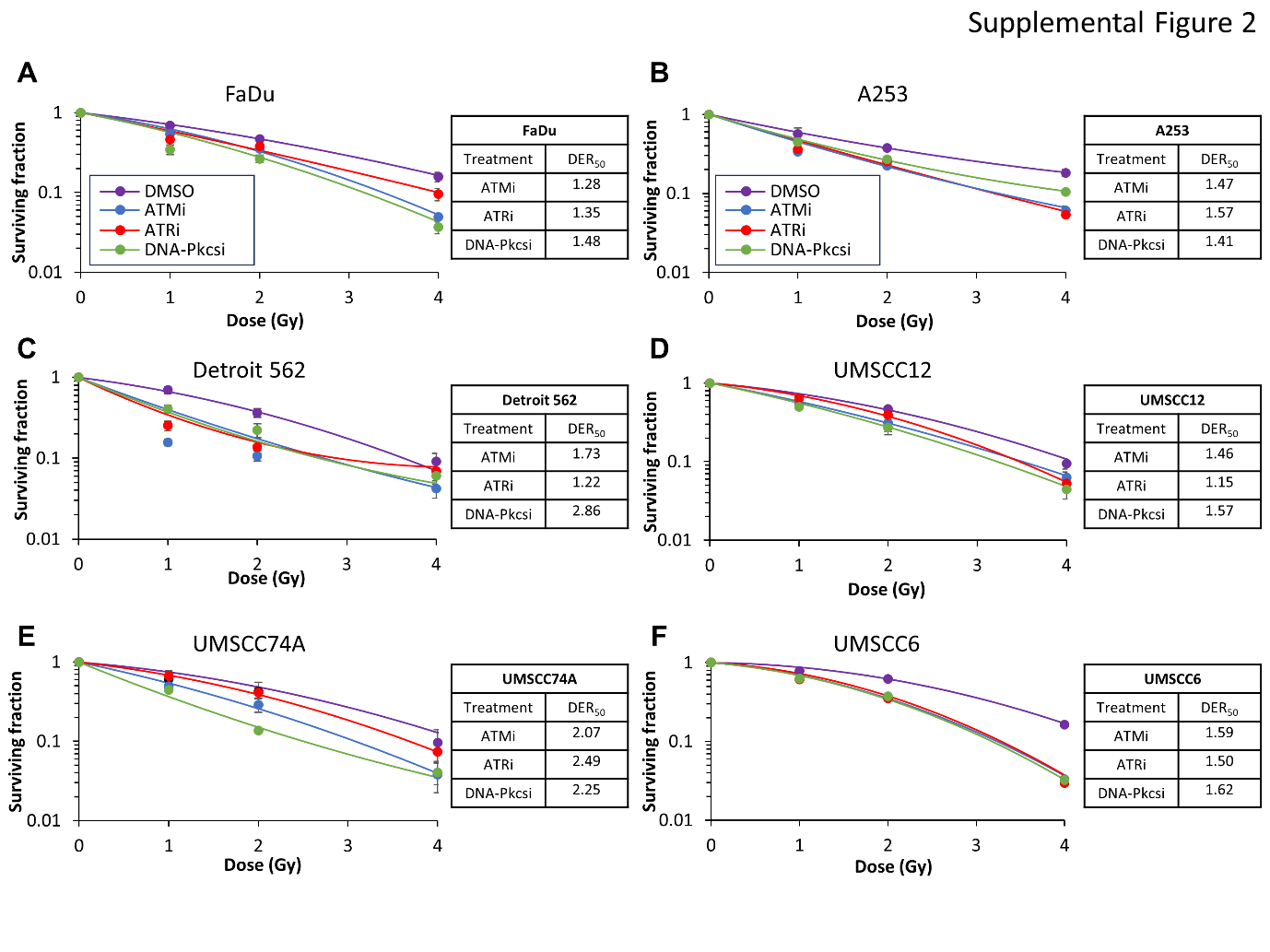
**

**Supplementary Figure 5. Inhibition of ATM, ATR and DNA-PKcs leads to increased radiosensitivity of HNSCC cells in response to X-ray radiation**. (A) FaDu, (B) A253, (C) Detroit 562, (D) UMSCC12, (E) UMSCC74A and (F) UMSCC6 cells were pretreated with inhibitors targeting ATM (10 nM), ATR (1 µM) and DNA-PKcs (1 µM) or DMSO as a vehicle only control, for 1 h prior to irradiation. Cells were then irradiated with increasing doses of X-rays, and clonogenic survival of cells was analysed from three biologically independent experiments. Shown is the data fitted according to the linear quadratic model, with dose enhancement ratios calculated at a surviving fraction of 0.5.


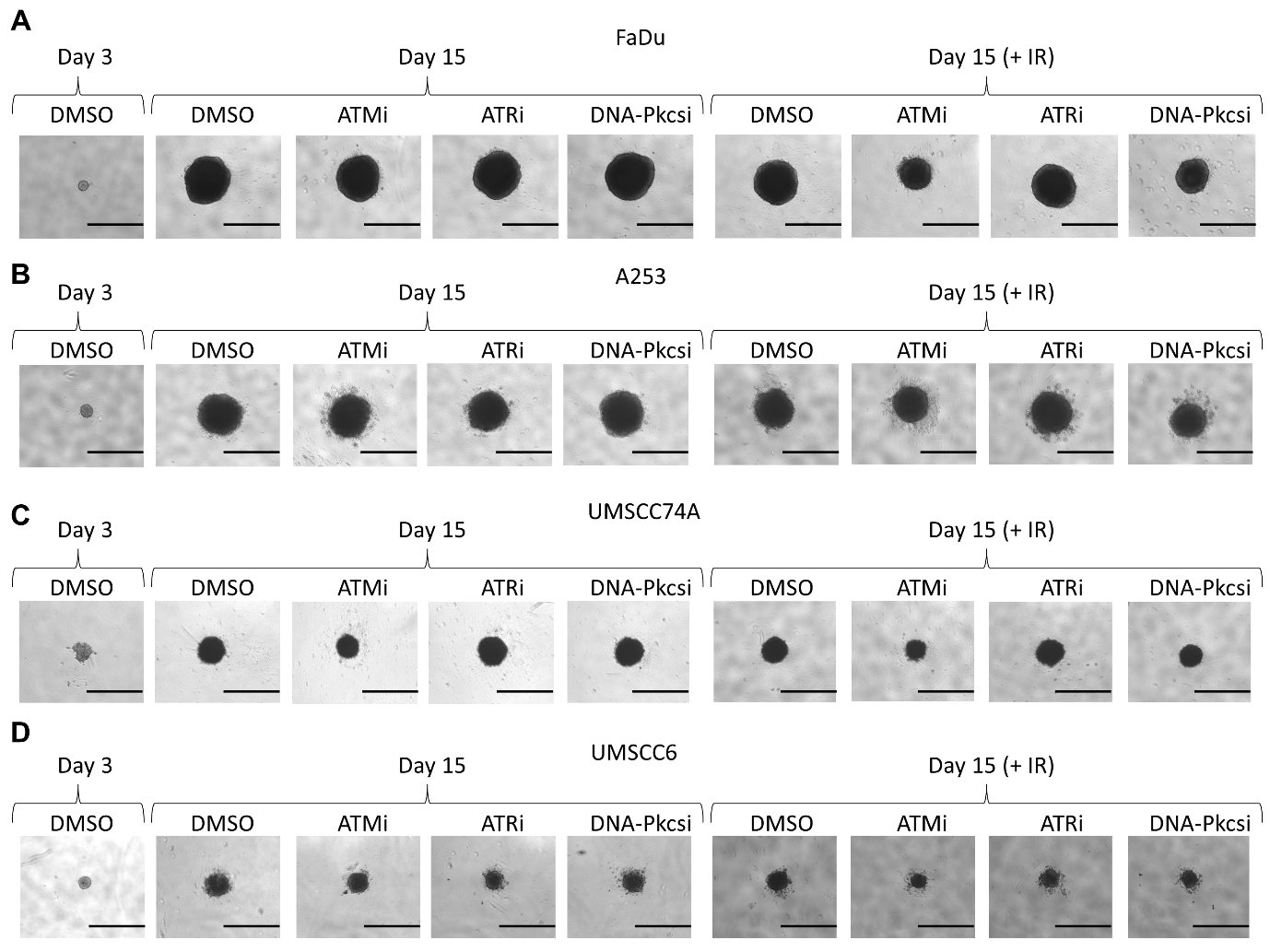


**Supplementary Figure 6. Inhibition of ATM, ATR and DNA-Pkcs leads to reduced growth of HNSCC 3D spheroids following X-ray radiation**. Spheroids were allowed to develop for 48 h in ultra-low attachment plates, and then treated with inhibitors targeting ATM, ATR, or DNA-Pkcs (all 0.2 µM) or DMSO as a vehicle only control for 1 h. Spheroids were then unirradiated, or irradiated with 1 Gy X-rays and growth of (A) FaDu, (B) A253, (C) UMSCC74A and (D) UMSCC6 spheroids measured by microscopy up to 15 days post-seeding. Shown are representative spheroid images, including the scale bar of 1000 µm.

**
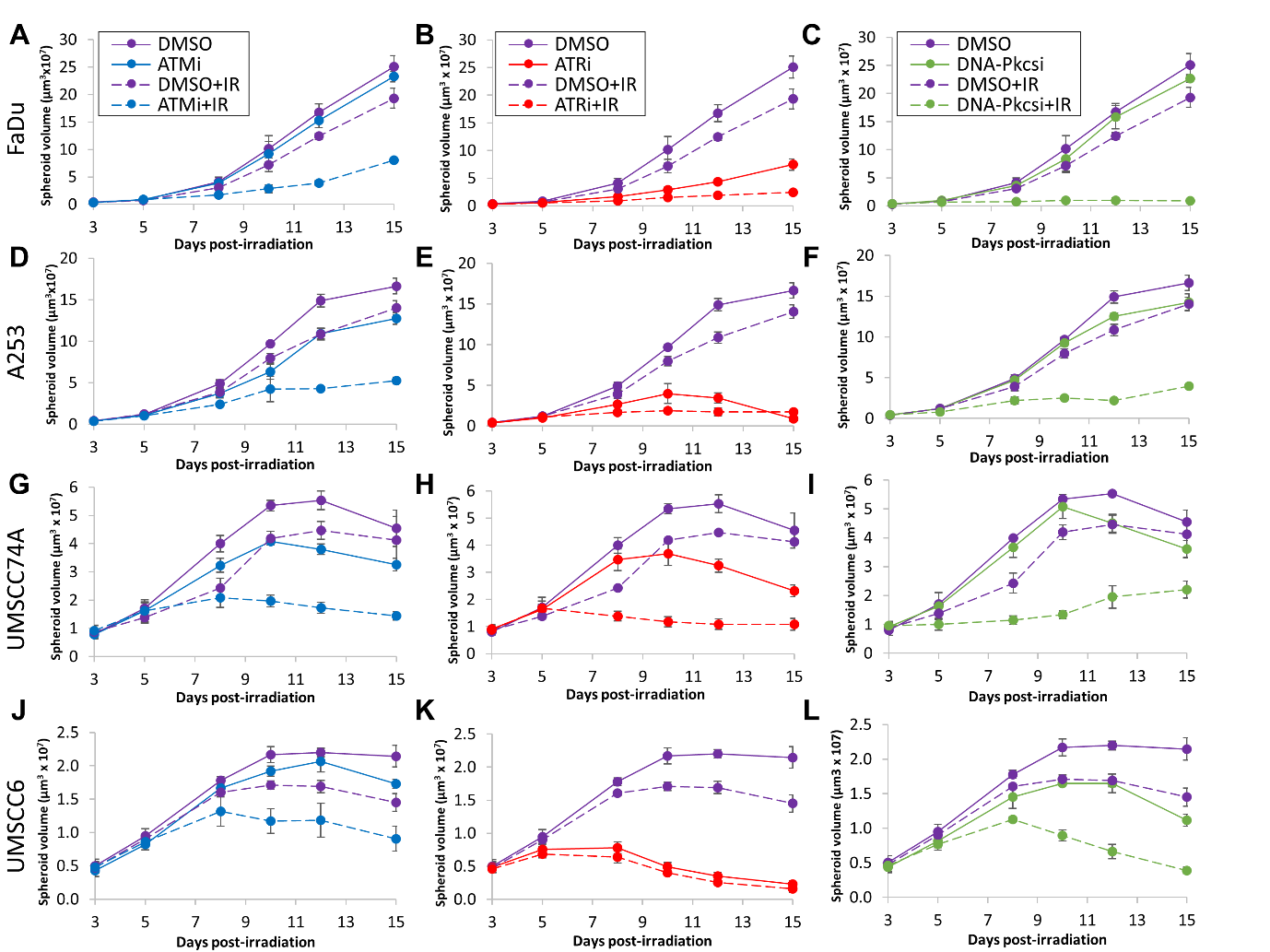
**

**Supplementary Figure 7. Inhibition of ATM, ATR and DNA-Pkcs leads to reduced growth of HNSCC 3D spheroids following X-ray radiation**. Spheroids were allowed to develop for 48 h in ultra-low attachment plates, and then treated with inhibitors targeting ATM, ATR, or DNA-Pkcs (all 1 µM) or DMSO as a vehicle only control for 1 h. Spheroids were then unirradiated, or irradiated with 1 Gy X-rays and growth of (A-C) FaDu, (D-F) A253, (G-I) UMSCC74A and (J-L) UMSCC6 spheroids measured by microscopy up to 15 days post-seeding. Shown is the mean spheroid volume±S.E., analysed from three biologically independent experiments.

**
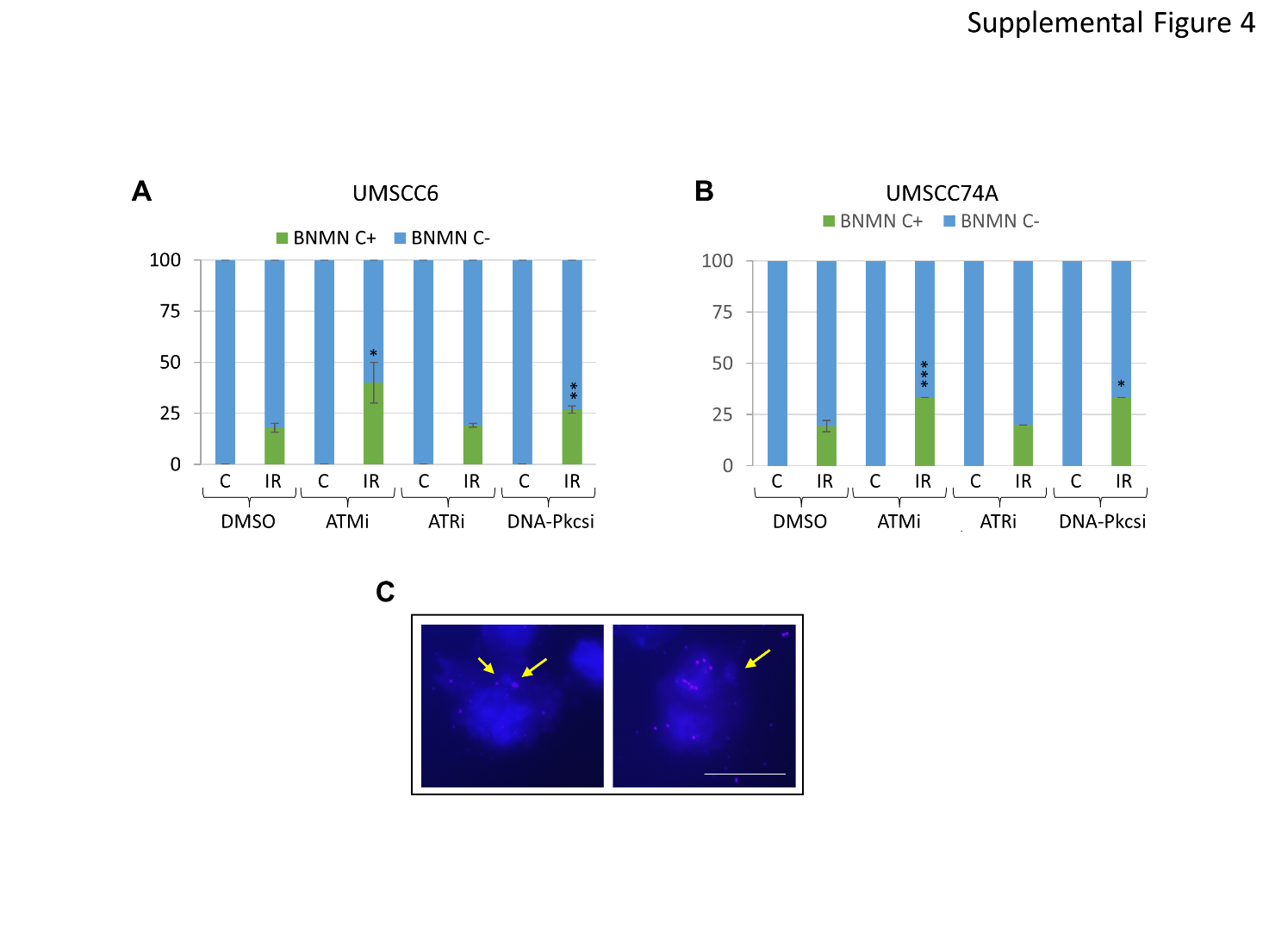
**

**Supplementary Figure 8. ATM, ATR and DNA-PKcs inhibition induces chromosomal aberrations in HNSCC cells after X-ray irradiation.** (A) UMSCC6 and (B) UMSCC74A, cells were treated with inhibitors targeting ATM (10 nM), ATR (1 µM) and DNA-PKcs (1 µM) or DMSO as a vehicle only control for 1 h prior to X-ray irradiation (2 Gy). Slides were then probed using pancentromeric probes, and the centromeric spots were analyzed by fluorescent microscopy. *p<0.05, **p<0.01, ***p<0.005 as analysed by a one sample *t*-test. (C) Representative image of BNMN C+ (left image) and BNMN C- (right image). Scale bar indicated is 10 µm.

**
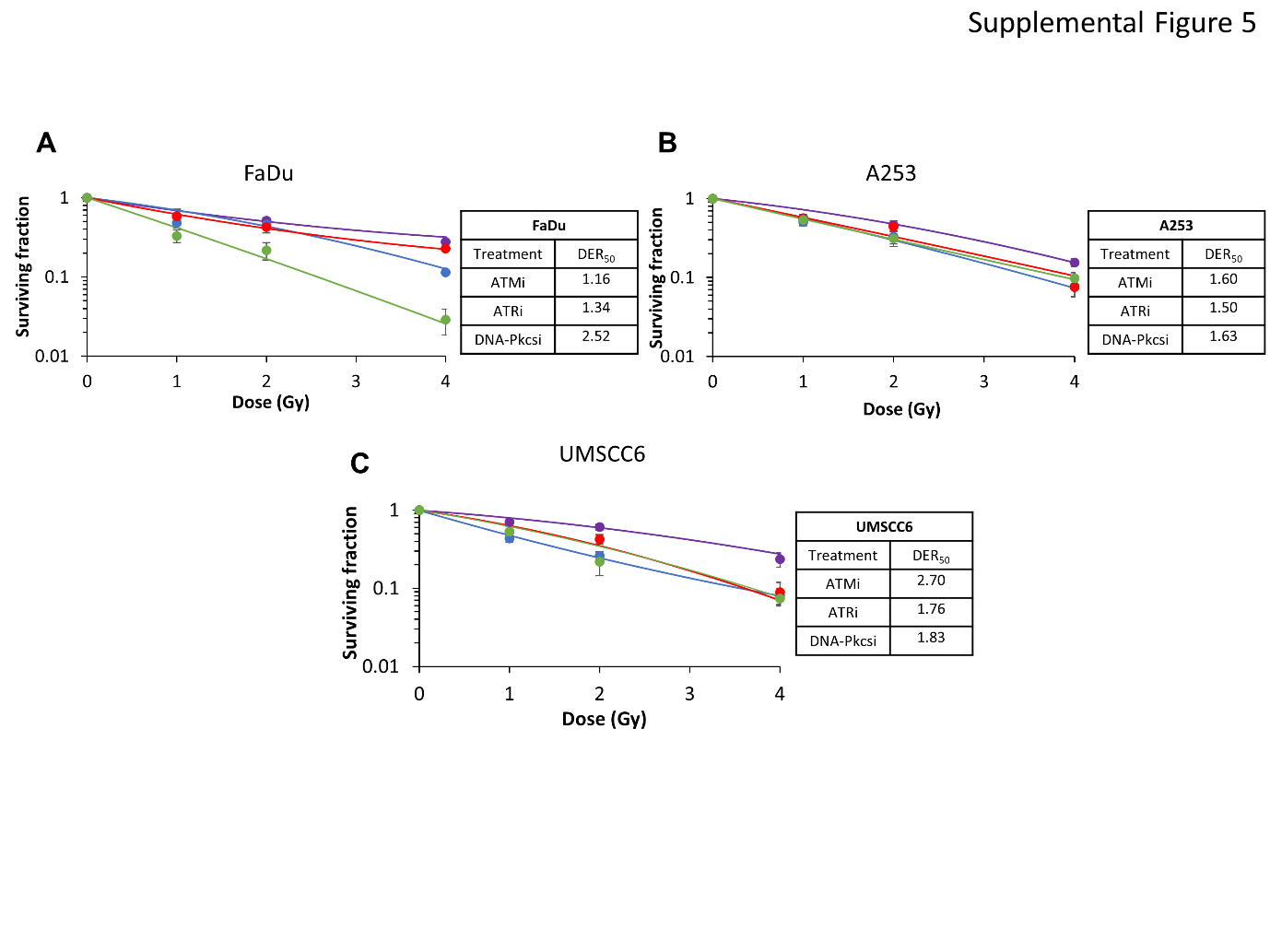
**

**Supplementary Figure 9. Inhibition of ATM, ATR and DNA-PKcs leads to increased radiosensitivity of HNSCC cells in response to PBT**. (A) FaDu, (B) A253 and (C) UMSCC6 cells were pretreated with inhibitors targeting ATM (10 nM), ATR (1 µM) and DNA-PKcs (1 µM) or DMSO as a vehicle only control, for 1 h prior to irradiation. Cells were then irradiated with increasing doses of PBT, and clonogenic survival of cells was analysed from three biologically independent experiments. Shown is the data fitted according to the linear quadratic model, with dose enhancement ratios calculated at a surviving fraction of 0.5.

**
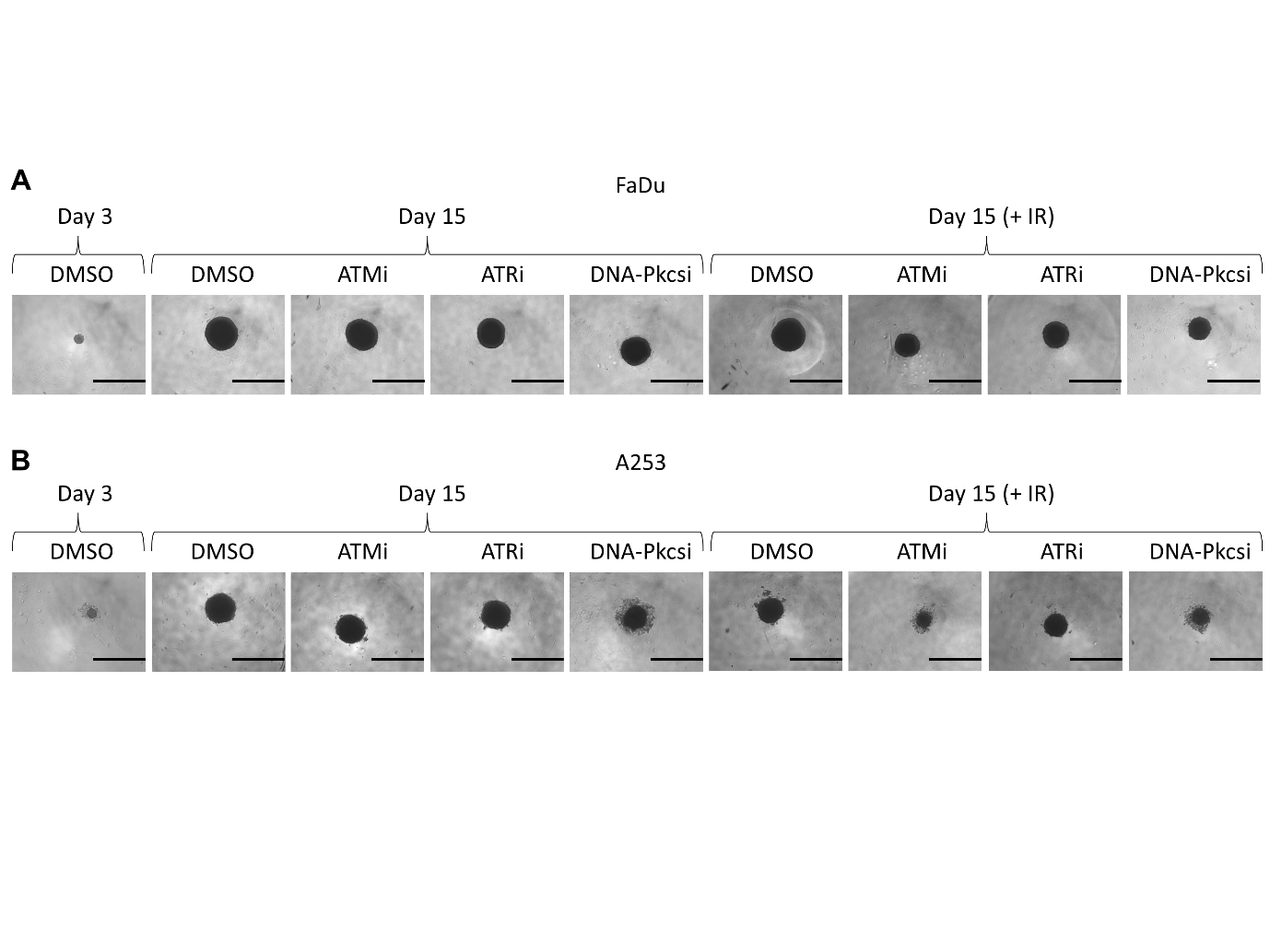
**

**Supplementary Figure 10. Inhibition of ATM, ATR and DNA-Pkcs leads to reduced growth of HNSCC 3D spheroids following PBT**. Spheroids were allowed to develop for 48 h in ultra-low attachment plates, and then treated with inhibitors targeting ATM, ATR, or DNA-Pkcs (all 0.2 µM) or DMSO as a vehicle only control for 1 h. Spheroids were then unirradiated, or irradiated with 1 Gy PBT and growth of (A) FaDu and (B) A253 spheroids measured by microscopy up to 15 days post-seeding. Shown are representative spheroid images, including the scale bar of 1000 µm.

**
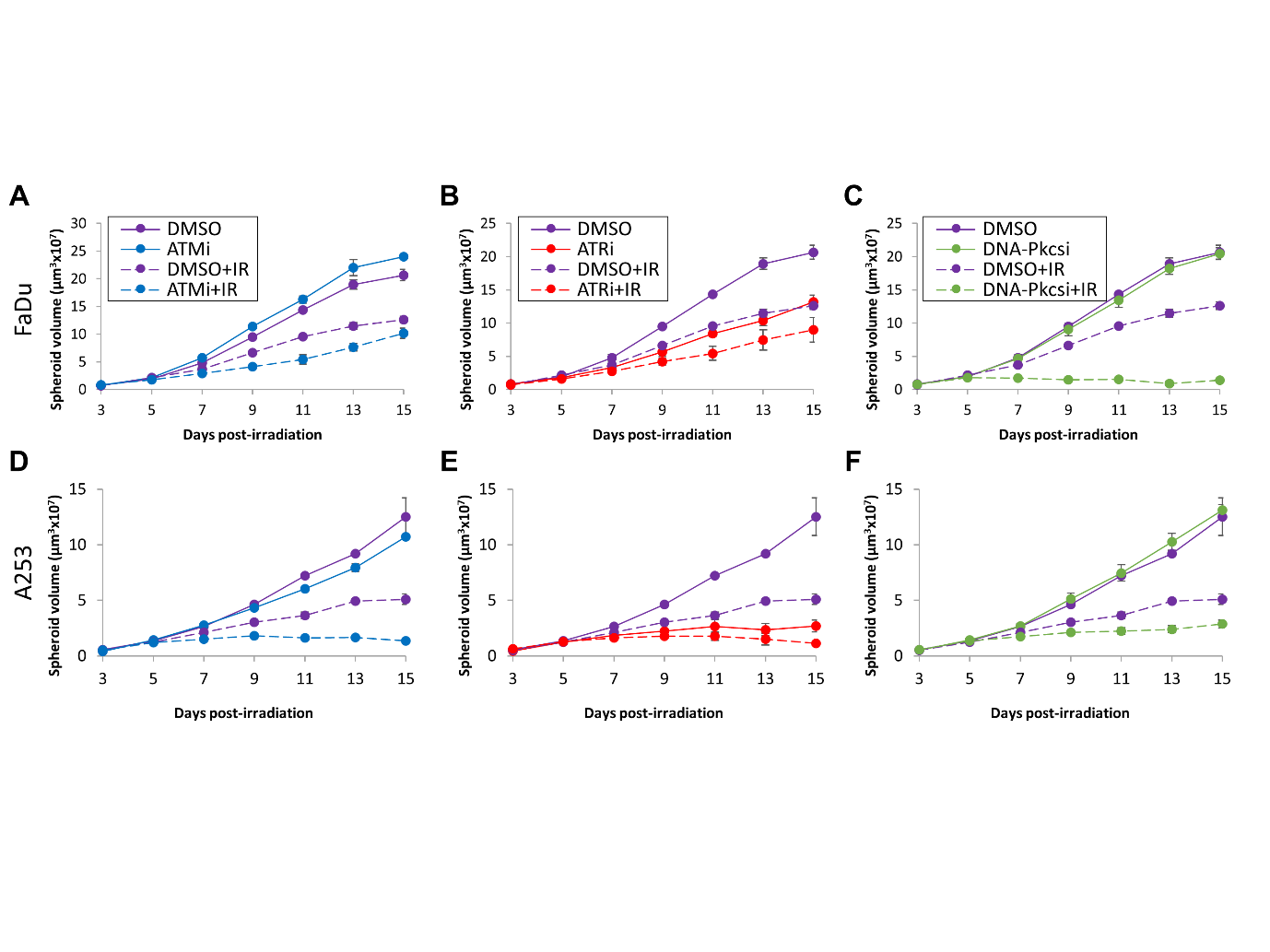
**

**Supplementary Figure 11. Inhibition of ATM, ATR and DNA-PKcs leads to increased radiosensitivity of HNSCC cells in response to PBT.** (A-C) FaDu and (D-F) A253 cells were were seeded in ultra-low attachment plates and spheroids were allowed to develop for 48 h. Spheroids were then treated with inhibitors targeting ATM, ATR, or DNA-PKcs (all 1 µM) or DMSO as a vehicle only control for 1 h, followed by irradiation with 1 Gy PBT (or left unirradiated as a control) and growth measured by microscopy up to 15 days post-seeding. Shown is the mean spheroid volume±S.E., analysed from three biologically independent experiments.
